# Hexaphenylditetrels – When Longer Bonds Provide Higher Stability

**DOI:** 10.1002/chem.202102271

**Published:** 2021-08-06

**Authors:** Lars Rummel, Jan M. Schümann, Peter R. Schreiner

**Affiliations:** ^1^ Institute of Organic Chemistry Justus Liebig University Heinrich-Buff-Ring 17 35392 Giessen Germany

**Keywords:** bond dissociation energy, bond strength, C−H-π-interactions, London dispersion, Pauli repulsion

## Abstract

We present a computational analysis of hexaphenylethane derivatives with heavier tetrels comprising the central bond. In stark contrast to parent hexaphenylethane, the heavier tetrel derivatives can readily be prepared. In order to determine the origin of their apparent thermodynamic stability against dissociation as compared to the carbon case, we employed local energy decomposition analysis (LED) and symmetry‐adapted perturbation theory (SAPT) at the DLPNO‐CCSD(T)/def2‐TZVP and sSAPT0/def2‐TZVP levels of theory. We identified London dispersion (LD) interactions as the decisive factor for the molecular stability of heavier tetrel derivatives. This stability is made possible owing to the longer (than C−C) central bonds that move the phenyl groups out of the heavily repulsive regime so they can optimally benefit from LD interactions.

## Graphical abstract

**Carbon‐Carbon bonds are exceptional** as demonstrated for the hexaphenylditetrels where attractive London dispersion interactions are the decisive factor for the thermodynamic stabilities of tetrels other than carbon. Structural and energetic comparisons show that even though hexaphenylethane displays the largest dispersion energy between the two molecular halves, it has remained elusive because of even larger Pauli repulsion of the phenyl moieties that are forced in close contact due to the C−C bond that is the shortest in this series.

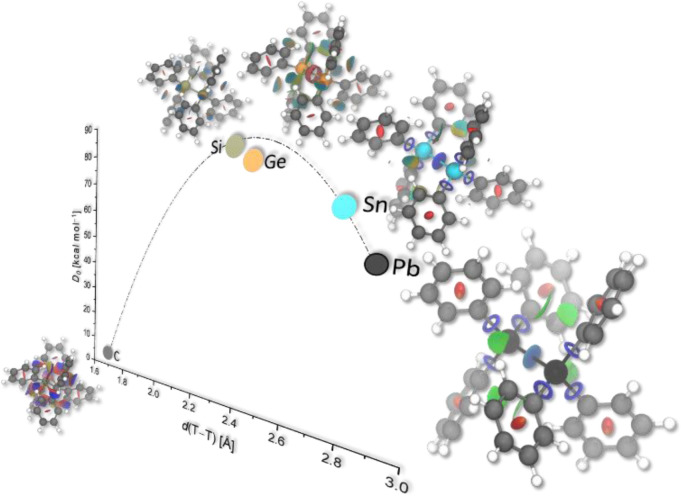

While long sought‐after hexaphenylethane^[1]^ (**1C**, Figure 1, the letter T designates the tetrel) remains elusive^[2]^ (trityl radicals dimerize in a head‐to‐tail fashion),^[3]^ its higher tetrel congeners with T=CSi,^[4]^ Si,^[5]^ Ge,^[6]^ Sn,^[7]^ and Pb^[8]^ have been known for a long time. What makes the latter stable under ambient conditions even though the higher tetrel‐tetrel single bond energies decrease rapidly as one goes down group 14?

**Figure 1:**
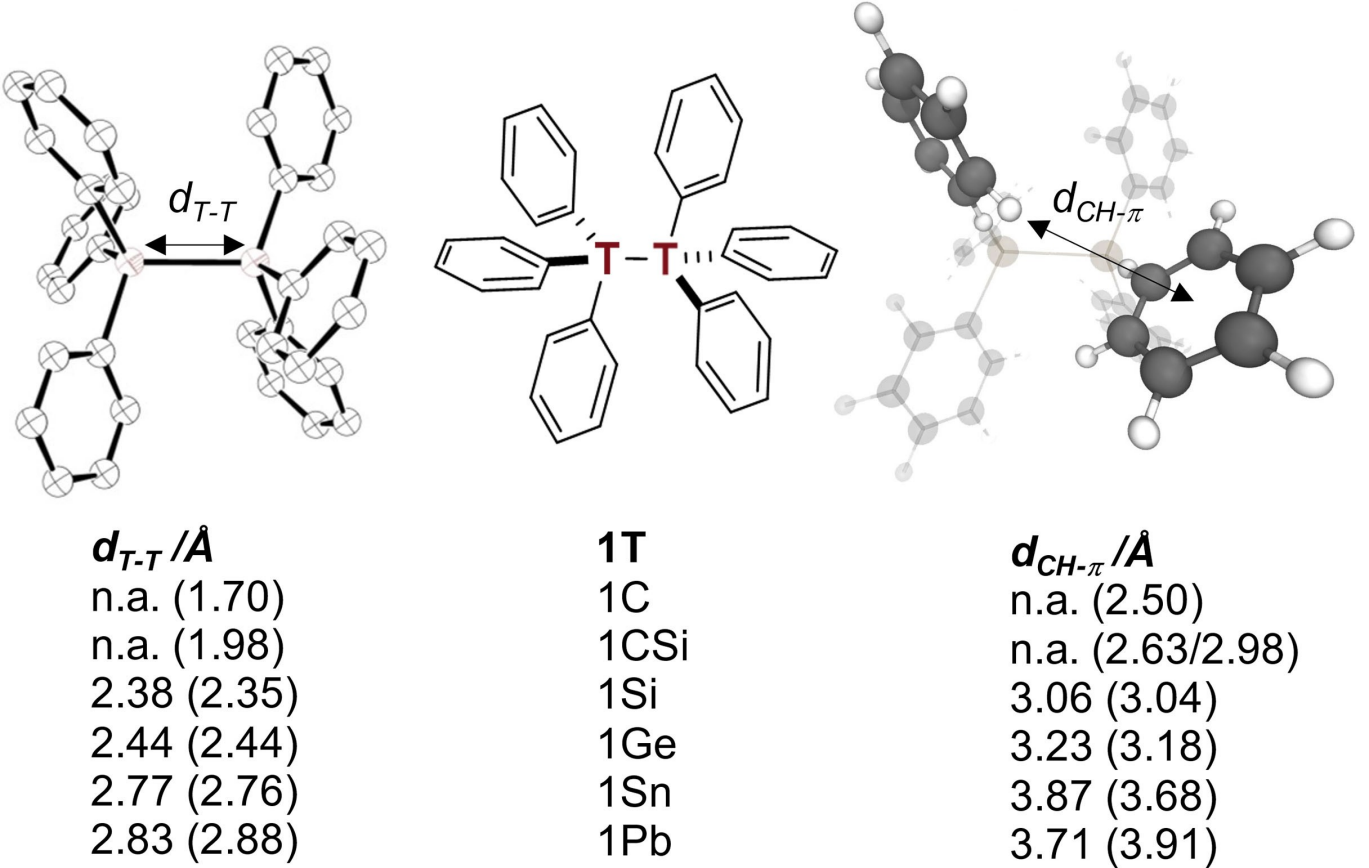
*S*_6_‐symmetric hexaphenylditetrel structure **1T** (center), X‐ray structure (left), and corresponding computed hexaphenylditetrel structure with highlighted *d*_CH−*π*_ contact at B3LYP‐D3(BJ)/def2‐TZVP (right). First numbers are experimental distances, numbers in parentheses correspond to the computations.

The Pb−Pb bond dissociation energy (BDE) of hexamethyldiplumbane is 22.5 kcal mol^−1^ lower than that of the central C−C bond in “hexamethylethane” (2,2,3,3‐tetramethylbutane, BDE=77.1±1.0 kcal mol^−1^),^[9]^ in line with the expectations of bond energies down a group in the periodic table.^[10]^ The opposite is observed for hexaphenylditetrels **1T** and parent **1C** has not been reported experimentally. Only some highly substituted derivatives utilizing dispersion energy donors^[11]^ (DED) such as *t*butyl groups in the all‐*meta* positions of **1C** can be observed,[^2b^, ^12^] thereby emphasizing the notion of stabilizing London dispersion (LD) interactions.^[13]^ As the higher tetrel derivatives do not need additional DED groups to be isolable, but intrinsically have higher T−T BDEs than **1C**, one asks what makes these compounds stable toward central T−T bond dissociation. Note that some derivatives with T=Sn are extraordinarily stable, even up to 235 °C.^[14]^ The first equilibrium measurement of a **1Sn** derivative was with the phenyl groups equipped with 2,4,6‐trimethyl and triethyl substituents. The onset of dissociation as measured through the presence of EPR signals of the “hetero‐Gomberg‐type” radicals was found at 180 and 100 °C for these derivatives, respectively.^[15]^

Even though there is no physical basis, there is a well‐accepted principle in organic chemistry that longer bonds are assumed to be weaker and therefore dissociate more easily.^[16]^ While this simple diatomic model‐derived concept has been investigated and confirmed for large series of molecules, it cannot explain the discrepancy in thermodynamic stabilities of the hexaphenylditetrels **1T**. Especially for large structures (i. e., far beyond diatomics), the transferability of this concept is questionable.^[17]^ Prominent examples are the phosphine‐metal dissociation energies of Grubbs catalysts with sterically demanding N‐heterocyclic carbene ligands^[18]^ and 2‐(1‐diamantyl)‐[121]tetramantane with a bond length of 1.71 Å but a sizeable BDE of around +36 kcal mol^−1^.^[19]^

As studies highlight that the noncovalent van‐der‐Waals benzene dimers are stabilized by LD interactions,^[20]^ we hypothesized that such interactions may be responsible for the stabilities of the higher **1T** structures as well. But why does this apparently not provide sufficient stabilization for **1C**?

We began our computational study with the crystal structure geometries for gas phase optimizations. Following the theoretical treatment of Rösel et al.^[2b]^ we utilized the well‐established B3LYP^[21]^ and M06‐2X^[22]^ functionals for direct comparisons with existing data and because they are commonly employed. Ahlrich's def2‐TZVP basis set^[23]^ was used for all computations. B3LYP was used with the Becke‐Johnson (BJ) damped dispersion D3 correction of Grimme et al.^[24]^ First and foremost, the optimized structures are in good agreement with the experimental structures (Figure 1 and Figures S2–S5, Table S10). All phenyl moieties are arranged in an off‐set T‐shape manner with CH−*π* contacts with the opposite trityl group. The computed dimerization energy of the triphenylmethyl radical is endergonic ($\Delta G_{\dim}^{298}$ = +11.8 kcal mol^−1^) and agrees with the results of previous studies.^[2b]^ Both the B3LYP‐D3(BJ) and M06‐2X results show the same trends. Due to a lack of experimental dissociation energies for the unsubstituted **1T**, we validated our method by comparing dissociation energies of H_3_T–TH_3_ as well as Me_3_T–TMe_3_ that agree well with experimental values within their error bounds (Tables S1–S3, Figure S1).

Whereas the carbon‐based hexaphenylditetrel readily dissociates into its monomers ($\Delta G_{\dim}^{298}$ >0), the higher tetrel derivatives all display $\Delta G_{\dim}^{298}$ <0 up to −70 kcal mol^−1^ (Figure 2). The reason behind the dissociation of **1C** can only be explained by Pauli (exchange) repulsion that has a very steep distance dependence, outweighing LD interactions, in line with the notion of excessive steric hindrance. Due to close intramolecular contacts of the aromatic moieties, hexaphenylethane **1C** cannot persist at 298 K (the computed shortest contact *d*_CH−*π*_ in **1C** is around 2.5 Å). However, as higher tetrels display significantly longer central bonds, this leads to an increase of the CH−*π* contact distances (the computed *d*_CH−*π*_ in **1Si** is around 3.1 Å, Figure 1). In comparison, the CH−*π* distance in the crystal lattice of benzene at 270 K is around 2.9 Å.^[25]^

**Figure 2:**
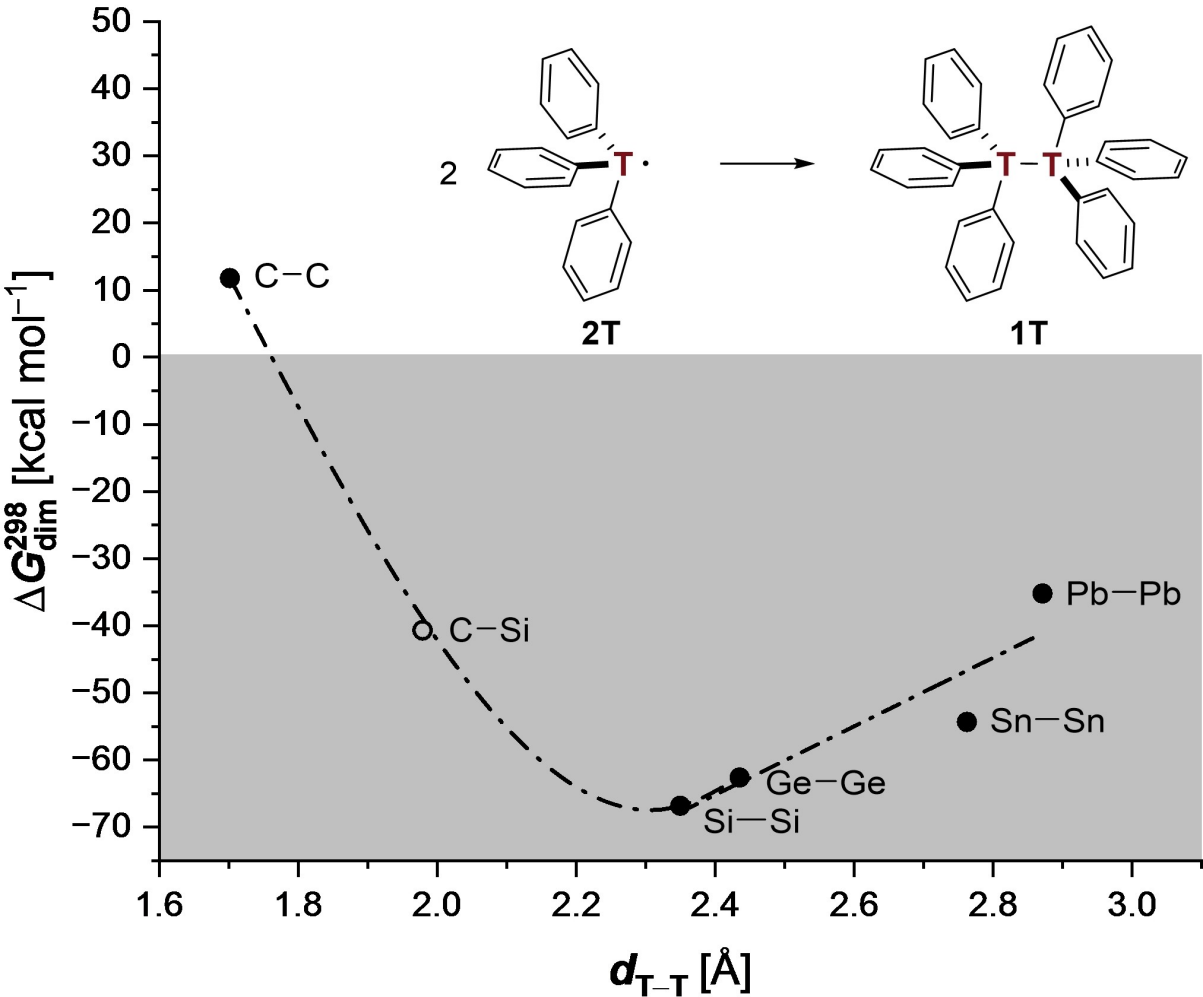
Correlation of distance ***d***_**T−T**_ [Å] of the central tetrel bond with the computed Gibbs free energies $\Delta G_{\dim}^{298}$ [kcal mol^−1^] for the depicted dimerization reaction. Computations at the B3LYP‐D3(BJ)/def2‐TZVP level of theory. The dashed line is used to guide the eye.

To investigate the origin of the somewhat counterintuitive stabilities of the higher tetrel congeners, we visualized all intramolecular interactions using non‐covalent interaction plots (NCI plots, Figure 3) for T=C vs. Pb.^[26]^ Hereby, strongly attractive and repulsive interactions are visualized as blue and red isosurfaces, respectively. Green areas indicate weak molecular contacts predominantly evoked by LD interactions.

**Figure 3:**
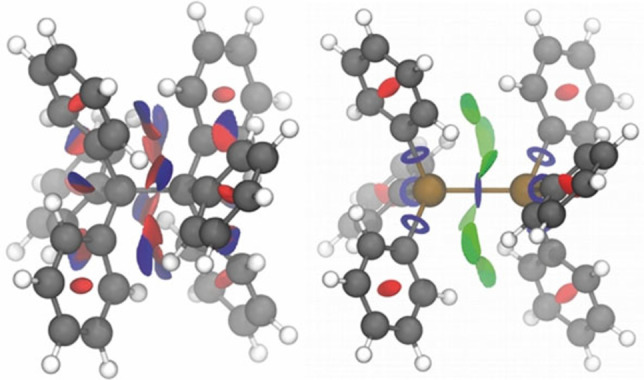
Non‐covalent interaction (NCI) plots of hexaphenylethane **1C** (left) and the hexaphenyldiplumbane **1Pb** compound (right) at the B3LYP‐D3(BJ)/def2‐TZVP level of theory. Isosurfaces are colored on a blue‐green‐red scale according to an isovalue s(*ρ*) of 0.2, ranging from *ρ*(r)=−2 a.u. to +2 a.u. Blue indicates strong attractive interactions, green corresponds to weak NCI, and red indicates strong repulsion.

A comparison of the NCI‐plots reveals strong repulsions (red) and strong attractions (blue) but no “weak” interactions (green) in **1C** between the two molecular halves. The opposite is observed for **1Pb** (with the same drawing cut‐offs) that clearly shows a green isosurface orthogonal to the central bond, emanating from the phenyl substituents.

Another approach for assessing the LD contributions is through splitting the central tetrel bond and analyzing the interactions between the two resulting fragments via a Local Energy Decomposition (LED) analysis^[27]^ as implemented in ORCA (Version 4.1.2).^[28]^ As a consequence of this approach, two radical fragments interact at short range, resulting in large electrostatic interactions. Hence, in this analysis we focus only on the magnitude of the LD interactions evoked by three phenyl‐phenyl CH−*π* contacts (Figure 4). According to this analysis, **1C** benefits from the highest LD contribution, while all higher congeners are LD‐stabilized by a remarkably similar amount around 20±5 kcal mol^−1^ for T≠C. That is, the instability of **1C** is not due to an insufficient LD stabilization but must lie in the massive growth of steric repulsion at short distance (see above). Vice versa, the lengthening of the central T−T bonds reduces Pauli repulsion more than dispersion so that an overall stabilization results.

**Figure 4:**
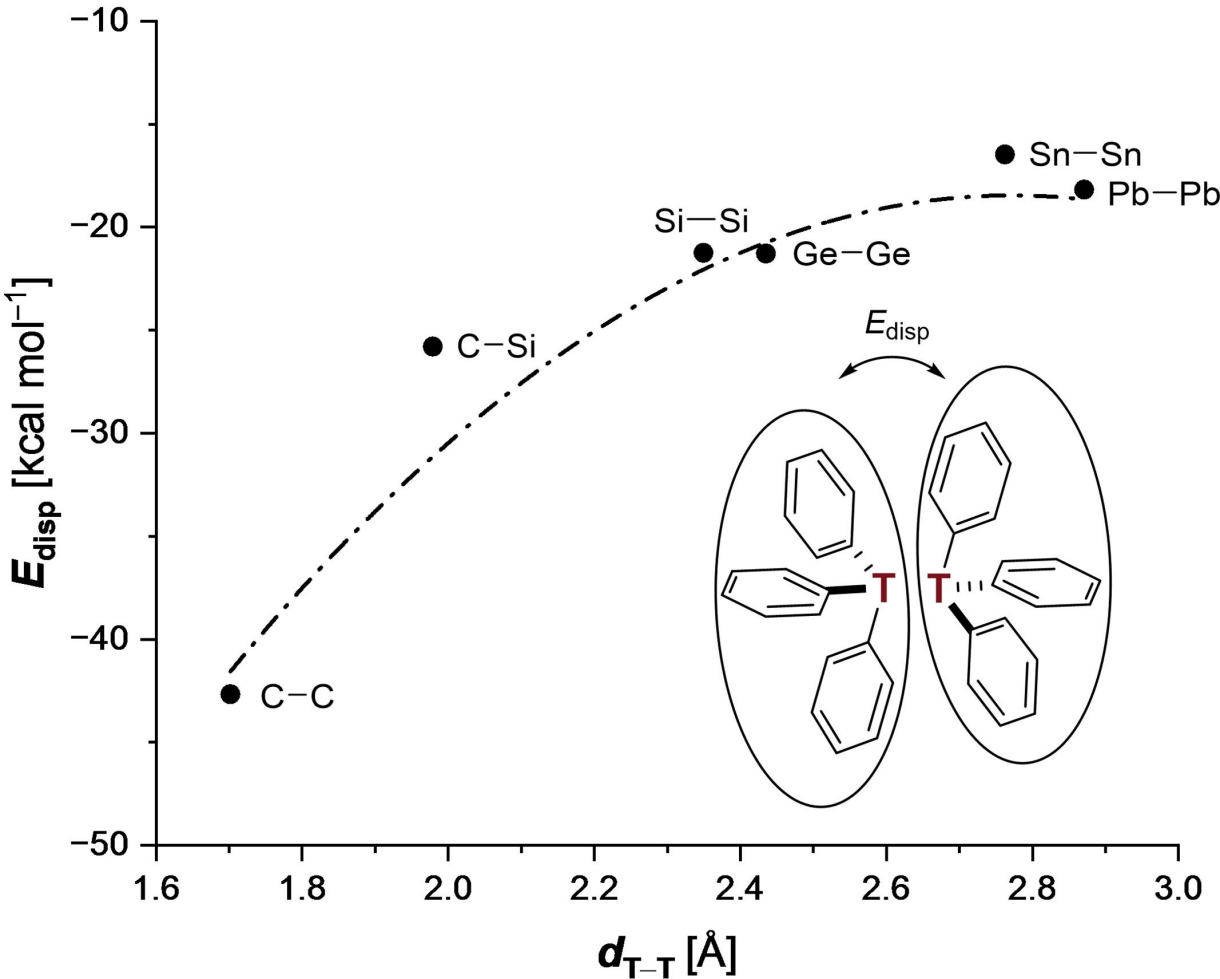
LED analysis of two trityl monomer singlet radicals in their dimer geometry at DLPNO‐CCSD(T)/def2‐TZVP//B3LYP‐D3(BJ)/def2‐TZVP. The dashed line is used to guide the eye.

In addition to the LED analysis, we utilized a homodesmotic equation^[29]^ (Figure 5) to determine the overall relative thermodynamic stabilities of **1T**. Thereby, we aimed at isolating the amount of LD due to the three pairwise phenyl‐phenyl contacts excluding the central tetrel interactions through calculating ΔΔ*E*_disp_=Δ*G* (B3LYP‐D3(BJ))‐Δ*G* (B3LYP).

**Figure 5:**
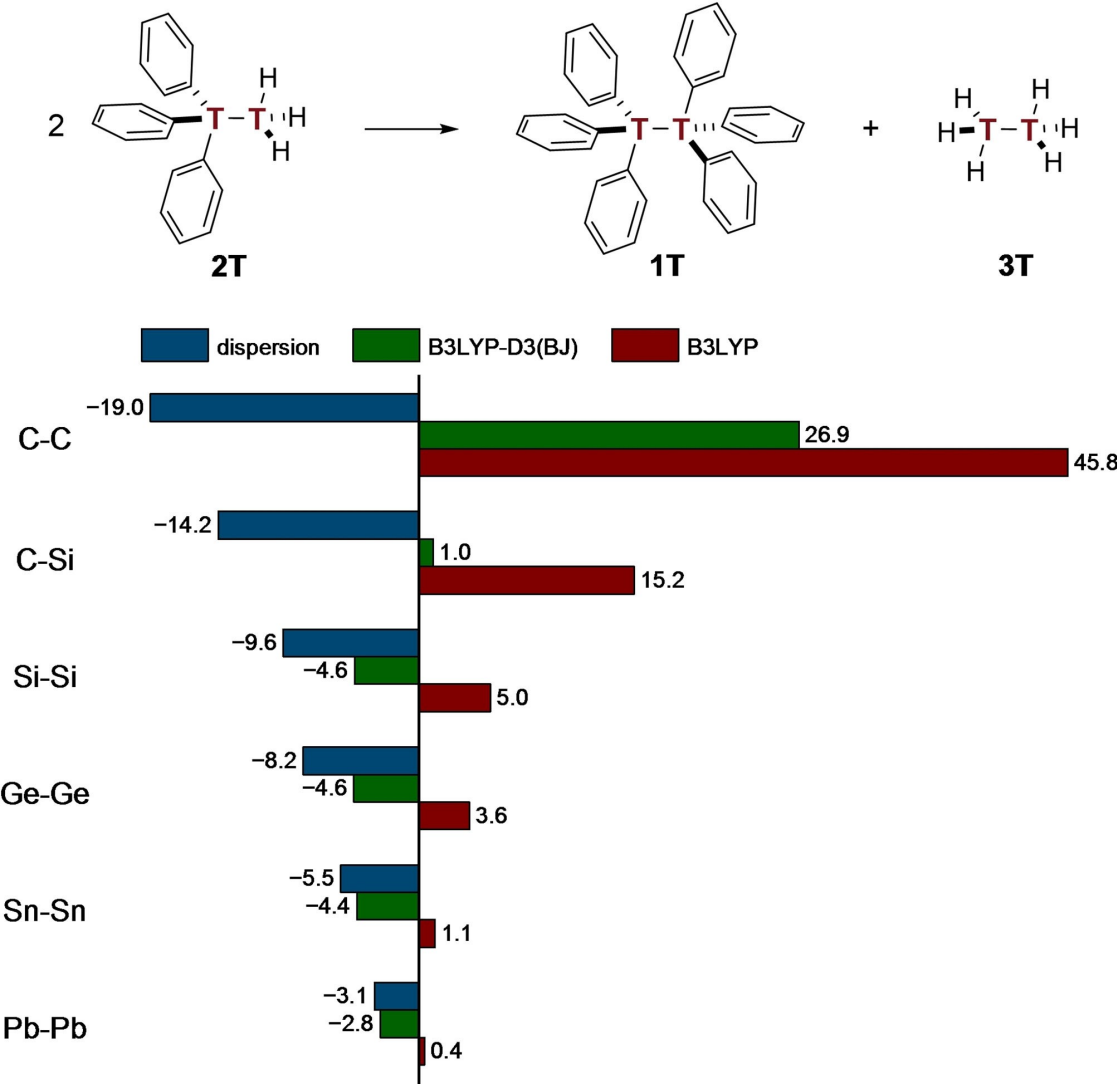
Homodesmotic equation with free energies (Δ*G*_298_) given in kcal mol^−1^ at the B3LYP‐D3(BJ)/def2‐TZVP level of theory.

The DFT computations not including LD suggest that the presence of all six phenyl groups within one molecule (**1T**) is highly unfavorable relative to distributing them across two triphenylditetrels **2T**. This picture would support the widespread notion of the predominance of steric hindrance. The elongation of the central tetrel bond entails a rapid decrease in repulsive energy from ∼46 kcal mol^−1^ in **1C** to only 0.4 kcal mol^−1^ in **1Pb**. Additionally, inclusion of LD, estimated from the value of the D3 correction, stabilizes all structures. Even though **1C** is stabilized most, LD cannot outbalance the strong repulsions, leading to an overall thermodynamically unstable structure. As repulsion reduces upon central bond elongation, all other tetrel derivatives beyond **1C** are stabilized overall. Whereas mixed **1CSi** is thermo‐neutral in terms of LD and electron‐electron repulsion, higher tetrel derivatives are stable due to LD that falls off less rapidly than Pauli repulsion. Consequently, LD interactions are most effective in the tetrel derivative with the longest bond (**1Pb**) where the total energy for this equation is comprised of 90 % LD interactions.

As most recently demonstrated by Herbert and Carter‐Fenk,^[20c]^ LD interactions and Pauli repulsion are the dominant factor in the noncovalent dimerization process of two benzene molecules, with the electrostatic component essentially being sidelined.[^20a^, ^20b^, ^27a^, ^30^] Within the series of hexaphenylditetrels the phenyl moieties adopt an off‐set T‐shaped geometry to optimized these two dominant interactions. This supports our findings since **1Si** is the most stable hexaphenylditetrel with an off‐set CH−*π* distance of 3.1 Å. In order to qualitatively evaluate the dispersion energy deriving from phenyl moieties, we also employed a symmetry‐adapted perturbation theory (SAPT) analysis.^[31]^ The scaled protocol was utilized to improve performance of the SAPT computations according to Parker et al.^[32]^ Hereby, we focus on the interaction between benzene dimers.^[33]^ We took the B3LYP‐D3(BJ)/def2‐TZVP optimized geometries, removed the tetrels, and saturated the resulting phenyl radicals with hydrogen atoms in order to avoid open‐shell configurations^[34]^ (Figure 6). The total interaction energy (black) shows an energy minimum at a central bond distance *d*_Si–Si_ of around 2.3 Å. The carbon derivative with a *d*_C−C_ of 1.7 Å is again the only thermodynamically unstable **1T** due to the large Pauli exchange repulsion term (red). All other structures are situated within the attractive part of the diagram. While LD interactions (green) are the main attractive component, electrostatics (blue) as well as induction (brown) also favor the dimerization process.

**Figure 6:**
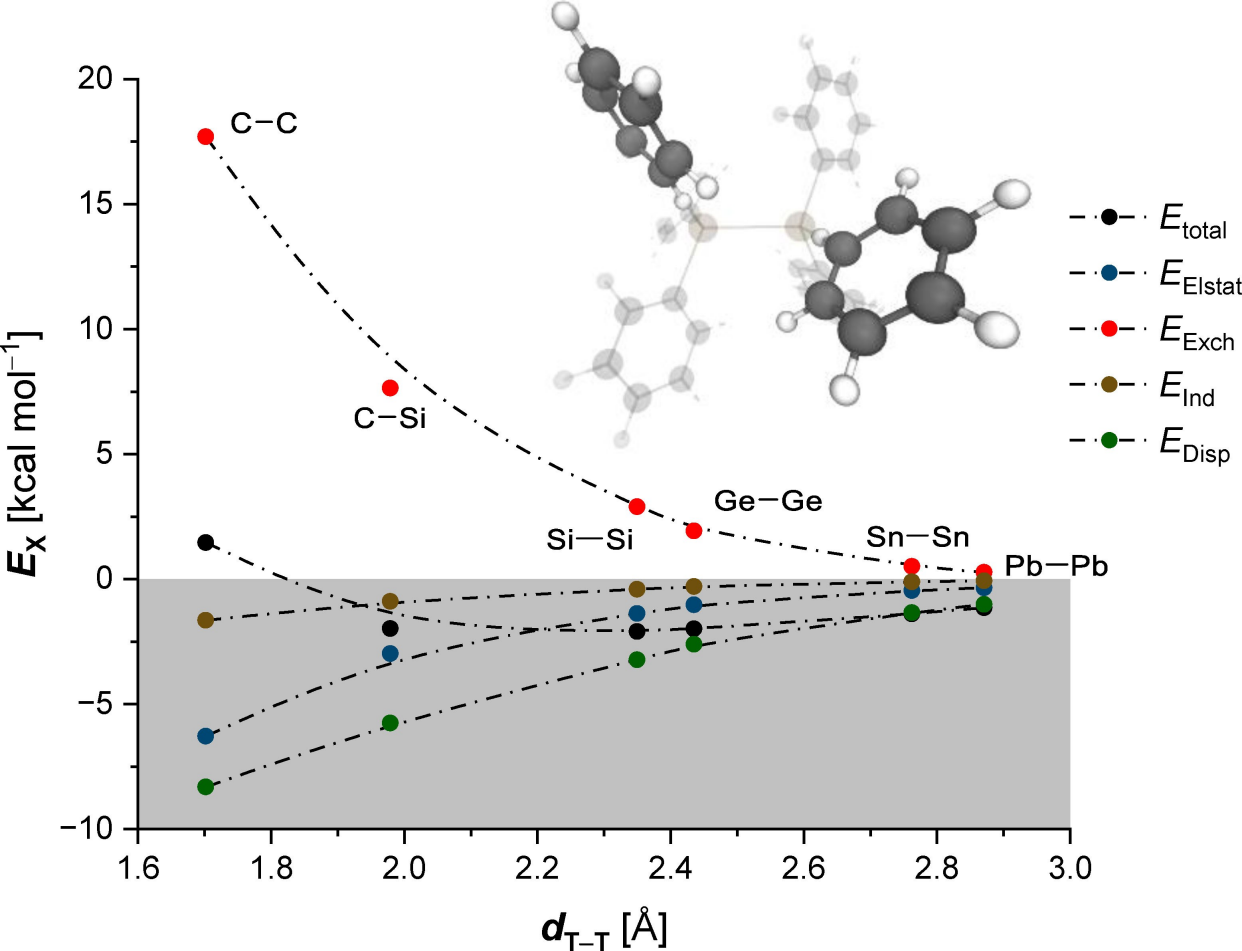
sSAPT analysis of two benzene monomers in geometry of the hexaphenylditetrels, *d*_T−T_ corresponds to the central tetrel bond. Computations at the sSAPT0/def2‐TZVP level of theory.

Our findings utilizing various interaction analyses and a homodesmotic equation are well in line with the conceptually simple but very useful *r* ^−12^ repulsive and *r* ^−6^ LD^[13a]^ attractive (12,6)‐Lennard‐Jones type potential of the noncovalent interaction distance. The much steeper repulsive potential may have led to the general notion in structural chemistry that repulsion may be more important overall, which is not true. As a consequence, hexaphenyldisilane (**1Si**) is the most stable parent hexaphenylditetrel derivative.

As we demonstrate here, there is a fine interplay of attraction and repulsion in molecular structures; naturally, that is why they are called “equilibrium structures.” As repulsion decreases rapidly with distance, LD is the most important stabilizing factor. The often invoked principle that longer bonds are to be weaker^[16]^ does not have to be true^[35]^ in the presence of additional interactions around the bonds in question. In the cases shown here this means that depending on the length of the central tetrel bond the phenyl groups can have a stabilizing or destabilizing effect on the structures. Hence, the high stability of the compounds with longer bonds is made possible through the assistance of LD interactions of the phenyl groups.

## Conflict of interest

The authors declare no conflict of interest.

## Supporting information

As a service to our authors and readers, this journal provides supporting information supplied by the authors. Such materials are peer reviewed and may be re‐organized for online delivery, but are not copy‐edited or typeset. Technical support issues arising from supporting information (other than missing files) should be addressed to the authors.

Supporting Information

Supporting Information
