# Supplementary material for: Hexaphenylditetrels – When Longer Bonds Provide Higher Stability
Source: Chemistry. 2021 Aug 6;27(55):13699–702. doi: 10.1002/chem.202102271 (PMC8518818; doi:10.1002/chem.202102271)
Supplement: Supplementary file 1 — Supporting Information [file CHEM-27-13699-s002.pdf]

Chemistry–A European Journal

Supporting Information

Hexaphenylditetrels – When Longer Bonds Provide Higher Stability

Lars Rummel[+], Jan M. Schümann[+], and Peter R. Schreiner*

Table of Contents

1. Computational Methods S2
2. Bond Lengths in Theory and Experiment S4
   2.1. Comparison of Dissociation Energies with the Literature............ S4
   2.2. Dissociation Energies of Hexaphenyltetrels (M06-2X, B3LYP)... S6
   2.3. Comparison of Crystal Structures and Gas Phase Computations S10
3. Local Energy Decomposition (LED) analysis.................................. S14
4. SAPT analysis S15
5. Discussion of LD assessments... S16
6. Cartesian Coordinates S17
   6.1. Ph_3T-TPh_3 Structures S17
   6.2. Ph_3T Radical Structures S26
   6.3. Me_3T-TMe_3 Structures S31
   6.4. Me_3T Radical Structures S33
   6.5. H_3T-TH_3 Structures S34
   6.6. H_3T Radical Structures S35
7. Additional Literature S36

1. Computational Methods

All geometry optimizations and single-point energy computations were performed with Gaussian16.[1] As a starting point for geometry optimizations the coordinates of the molecular crystal structures were utilized. Optimizations and energy computations were performed in the gas phase with the B3LYP[2] and M06-2X[3] functionals. The B3LYP computations were amended with the Becke-Johnson damped dispersion correction according to Grimme *et al.*.[4] The def2-TZVP basis set according to Ahlrich *et al.*[5] was found to be most suitable for all atoms present within the molecules studied here but comparisons with a mixed basis set consisting of SDD (for Sn and Pb) and cc-pVDZ[6] (for H, C, Si and Ge) were also made. All homodimers were restricted to S_6 symmetry, whereas the mixed tetrel dimer as well as the monomeric structures, were computed in C_3 symmetry. All optimized structures, obtained as described before, are located on their respective minimum of the energy hypersurfaces. Only hexaphenyldiplumbane showed very low imaginary frequencies ($< 10i\,\mathrm{cm}^{-1}$) which are due to insignificant numerical deficiencies. The optimized geometries of all molecules generally agree with the crystal structures reported in the literature (see Section 2.3 in SI). For further validation, we also computed the BDEs of H_3T-TH_3 and Me_3T-TMe_3.

Visualizations of non-covalent interactions (NCIs) were plotted as a reduced density gradient in regions of low electron density. The B3LYP-D3(BJ)/def2-TZVP optimized geometries were utilized for visualizing the non-covalent interactions. All plots were generated with NCIPLOT[7] and VMD.[8] The density cut-off of the reduced density gradient (ρ(r) = –0.2 a.u. to +0.2 a.u.) and the color scale data range (–2 a.u. to +2 a.u.) were kept consistent for all NCI-plots. Thereby, red isosurfaces indicate strongly repulsive interactions, green isosurfaces correspond to weak non-covalent contacts, and blue isosurfaces indicate strongly attractive interactions.

Local energy decomposition analyses (LEDs) were performed according to Neese *et al.*.[9] For this purpose ORCA 4.1.2[10] was employed as follows. The B3LYP-D3(BJ)/def2-TZVP optimized geometries were used as starting points for additional single-point energy computations at DLPNO-CCSD(T) using TightPNO (pair natural orbitals) settings. Again, the def2-TZVP basis set and its matching auxiliary basis set were utilized; the Pipek-Mezey localization scheme was utilized for PNOs.

Homodesmotic (error-balancing) equations[11] were performed to estimate the strength of the LD interactions. All structures were optimized in the gas phase using B3LYP/def2-TZVP including (GD3BJ) and excluding LD interactions. The dispersion energy was estimated according to the following equation:

$$E_{\mathrm{disp}} = \sum E(\text{product})\text{-D3(BJ)} - \sum E(\text{starting material})\text{-D3(BJ)} - \sum E(\text{product}) - \sum E(\text{starting material})$$

Finally, a scaled Symmetry-Adapted Perturbation Theory (sSAPT)[12] analysis was performed using the PSI4 program[13] using the B3LYP-D3(BJ)/def2-TZVP optimized geometries. The scaled protocol was employed to improve the performance of the SAPT computations according to Parker *et al.*.[14] In order to isolate the interaction between two phenyl moieties, all other atoms were removed and both phenyl radicals were terminated with a hydrogen radical.

2. Bond Lengths in Theory and Experiment

2.1. Comparison of Dissociation Energies with the Literature

All experimental $\boldsymbol{D_0^{298}}$ values are taken from the 97th edition Handbook of Chemistry and Physics[15] unless noted otherwise. This handbook refers to the comprehensive handbook of chemical bond energies.[16]

Table S1. Comparison of computed (B3LYP-D3(BJ)/def2-TZVP) ΔH_d^{298}, D_e, and D_0 with experimental D_0^{298} of $H_3T–TH_3$.

T	$d_{comp.}$(T–T) [Å]	$d_{exp.}$(T–T) [Å]	D_e [kcal mol⁻¹]	D_0 [kcal mol⁻¹]	ΔH_d^{298} [kcal mol⁻¹]	D_0^{298} (exp) [kcal mol⁻¹]
**C**	1.526	n.a.	93.6	84.2	85.8	90.2 (±0.2)
**Si**	2.344	n.a.	76.4	72.3	72.9	76.7 (±n.a.)
**Ge**	2.436	n.a.	70.8	67.0	67.4	n.a.
**Sn**	2.787	n.a.	59.6	56.5	56.5	n.a.
**Pb**	2.891	n.a.	49.2	45.9	45.8	n.a.
**C-Si**	1.876	n.a.	89.4	83.1	84.3	89.6 (±1.2)

Table S2: Comparison of computed (B3LYP-D3(BJ)/def2-TZVP) d(T-T), ΔH_d^{298}, D_e and D_0 with experimental D_0^{298} of Me_3T-TMe_3

T	$d_{comp.}$(T–T) [Å]	$d_{exp.}$(T–T) [Å]	D_e [kcal mol⁻¹]	D_0 [kcal mol⁻¹]	ΔH_d^{298} [kcal mol⁻¹]	D_0^{298} (exp) [kcal mol⁻¹]
**C**	1.581	n.a.	71.9	64.6	65.9	77.1 (±1.0)
**Si**	2.347	n.a.	77.5	75.5	75.0	80.5 (±1.0)[17]
**Ge**	2.443	n.a.	71.2	69.3	68.6	67.0 (±n.a.)
**Sn**	2.798	n.a.	58.9	57.2	56.3	61.6 (±3.5)
**Pb**	2.913	n.a.	46.6	44.4	43.5	54.6 (±n.a.)
**C-Si**	1.910	n.a.	79.2	75.1	75.4	n.a.

Table S3: Comparison of computed (B3LYP-D3(BJ)/def2-TZVP) ΔH_d^{298}, D_e and D_0 with experimental D_0^{298} of Ph_3T-TPh_3

T	$d_{comp.}$(T-T) [Å]	$d_{exp.}$(T-T) [Å]	D_e [kcal mol⁻¹]	D_0 [kcal mol⁻¹]	ΔH_d^{298} [kcal mol⁻¹]	D_0^{298} (exp) [kcal mol⁻¹]
**C**	1.702	n.a.	8.5	3.9	3.7	n.a. (±n.a.)
**Si**	2.350	2.462[18] (±n.a.)	83.0	80.7	79.9	88.0 (±n.a.)
**Ge**	2.435	2.437[19] (±n.a.)	77.4	75.7	74.7	n.a.
**Sn**	2.763	2.791[20] (±n.a.)	65.0	63.9	62.6	n.a.
**Pb**	2.871	2.830[21] (±n.a.)	51.9	50.3	49.1	n.a.
**C-Si**	2.883	n.a.	59.7	56.2	55.8	n.a.

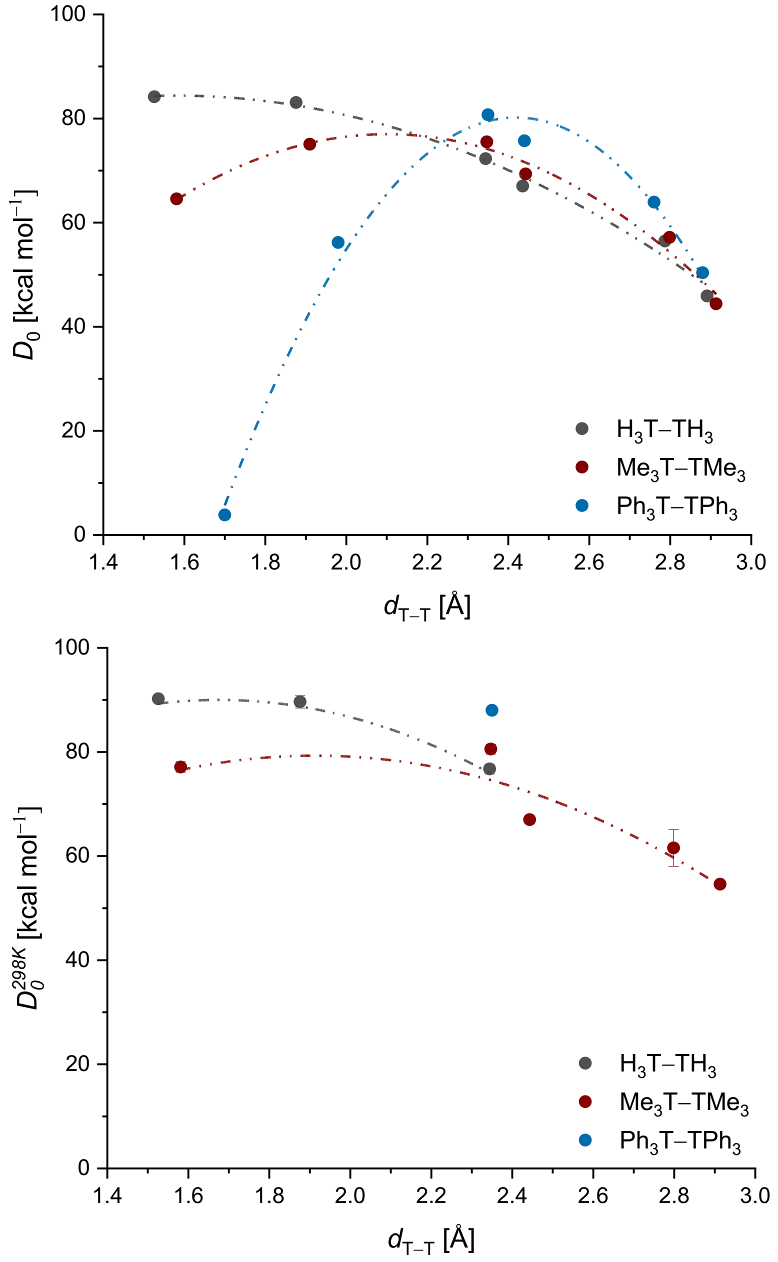

Figure S1: Visual comparison of computed D_0 (B3LYP-D3(BJ)/def2-TZVP) (top) and experimental data D^{298} (bottom) vs dT–T.

Figure S1 shows the same trends for the experimental and the computed dissociation energies of unsubstituted ditetrels and hexamethyl ditetrels; no experimental data of the corresponding hexaphenylditetrels, except for hexaphenyldisilane were found. However, the values cannot be compared directly with the computed results due to discrepancies of defining BDE's.[22]

2.2. Dissociation Energies of Hexaphenyltetrels (M06-2X, B3LYP)

Scheme S1: Dissociation of hexaphenylditetrels

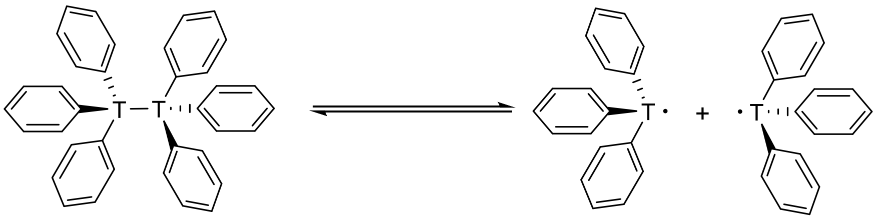

Table S4: Dissociation energies of hexaphenylethane

	D_e [kcal mol^{-1}]	D_0 [kcal mol^{-1}]	ΔH_d^{298} [kcal mol^{-1}]	ΔS_d^{298} [cal mol^{-1} K^{-1}]	$T\Delta S_d^{298}$ [kcal mol^{-1}]	ΔG_d^{298} [kcal mol^{-1}]
B3LYP cc-pVDZ/SDD	−18.2	−21.8	−21.6	52.4	15.6	−37.2
B3LYP-D3(BJ) cc-pVDZ/SDD	13.0	8.7	9.1	54.2	16.2	−7.0
M06-2X cc-pVDZ/SDD	16.9	12.8	13.3	54.4	16.2	−3.0
B3LYP def2-TZVP	−22.7	−26.8	−26.5	52.6	15.7	−42.1
B3LYP-D3(BJ) def2-TZVP	8.5	3.9	4.3	53.9	16.1	−11.8

Table S5: Dissociation energies of hexaphenyldisilane

	D_e [kcal mol^{-1}]	D_0 [kcal mol^{-1}]	ΔH_d^{298} [kcal mol^{-1}]	ΔS_d^{298} [cal mol^{-1} K^{-1}]	$T\Delta S_d^{298}$ [kcal mol^{-1}]	ΔG_d^{298} [kcal mol^{-1}]
B3LYP cc-pVDZ/SDD	63.2	61.5	61.1	43.9	13.1	48.0
B3LYP-D3(BJ) cc-pVDZ/SDD	83.5	81.5	81.2	44.9	13.4	67.8
M06-2X cc-pVDZ/SDD	79.2	77.5	77.3	48.1	14.3	63.0
B3LYP def2-TZVP	62.3	60.3	60.0	45.9	13.7	46.4
B3LYP-D3(BJ) def2-TZVP	83.0	80.7	80.5	45.9	13.7	66.8

Table S6: Dissociation energies of hexaphenyldigermane

	D_e [kcal mol^{-1}]	D_0 [kcal mol^{-1}]	ΔH_d^{298} [kcal mol^{-1}]	ΔS_d^{298} [cal mol^{-1} K^{-1}]	$T\Delta S_d^{298}$ [kcal mol^{-1}]	ΔG_d^{298} [kcal mol^{-1}]
B3LYP cc-pVDZ/SDD	59.5	58.1	57.6	41.9	12.5	45.1
B3LYP-D3(BJ) cc-pVDZ/SDD	78.3	76.7	76.3	41.9	12.5	63.8
M06-2X cc-pVDZ/SDD	68.5	67.3	66.9	42.4	12.6	54.3
B3LYP def2-TZVP	58.7	57.1	56.7	43.9	13.1	43.6
B3LYP-D3(BJ) def2-TZVP	77.4	75.7	75.3	42.5	12.7	62.6

Table S7: Dissociation energies of hexaphenyldistannane

	D_e [kcal mol^{-1}]	D_0 [kcal mol^{-1}]	ΔH_d^{298} [kcal mol^{-1}]	ΔS_d^{298} [cal mol^{-1} K^{-1}]	$T\Delta S_d^{298}$ [kcal mol^{-1}]	ΔG_d^{298} [kcal mol^{-1}]
B3LYP cc-pVDZ/SDD	51.4	50.6	49.8	30.3	9.0	40.8
B3LYP-D3(BJ) cc-pVDZ/SDD	64.8	64.2	66.1	62.2	18.6	47.6
M06-2X cc-pVDZ/SDD	64.7	63.7	63.0	34.8	10.4	52.6
B3LYP def2-TZVP	51.4	50.2	49.6	37.9	11.3	38.3
B3LYP-D3(BJ) def2-TZVP	65.0	63.9	63.2	29.7	8.8	54.3

Table S8: Dissociation energies of hexaphenyldiplumbane

	D_e [kcal mol^{-1}]	D_0 [kcal mol^{-1}]	ΔH_d^{298} [kcal mol^{-1}]	ΔS_d^{298} [cal mol^{-1} K^{-1}]	$T\Delta S_d^{298}$ [kcal mol^{-1}]	ΔG_d^{298} [kcal mol^{-1}]
B3LYP cc-pVDZ/SDD	41.4	40.3	39.6	30.9	9.2	30.3
B3LYP-D3(BJ) cc-pVDZ/SDD	53.7	52.7	53.6	51.4	15.3	38.3
M06-2X cc-pVDZ/SDD	53.7	52.6	51.9	28.1	8.4	43.5
B3LYP def2-TZVP	39.4	38.1	37.4	35.2	10.5	26.9
B3LYP-D3(BJ) def2-TZVP	51.9	50.4	49.7	30.4	9.1	40.7

Table S9: Dissociation energies of triphenyl(triphenylmethyl)silane

	D_e [kcal mol^{-1}]	D_0 [kcal mol^{-1}]	ΔH_d^{298} [kcal mol^{-1}]	ΔS_d^{298} [cal mol^{-1} K^{-1}]	$T\Delta S_d^{298}$ [kcal mol^{-1}]	ΔG_d^{298} [kcal mol^{-1}]
B3LYP cc-pVDZ/SDD	34.0	31.3	31.3	50.6	15.1	16.2
B3LYP-D3(BJ) cc-pVDZ/SDD	60.8	57.7	57.8	51.1	15.2	42.5
M06-2X cc-pVDZ/SDD	61.6	58.8	58.9	50.0	14.9	44.0
B3LYP def2-TZVP	32.4	29.4	29.5	51.6	15.4	14.1
B3LYP-D3(BJ) def2-TZVP	59.7	56.2	56.4	52.7	15.7	40.7

2.3. Comparison of Crystal Structures and Gas Phase Computations

In order to compare the crystal structures with computed structures, both were visualized (CPK model) and placed on top of each other. In all cases, the central bond lengths agree very well. Only the phenyl moieties tilt differently in the solid vs. the gas phase.

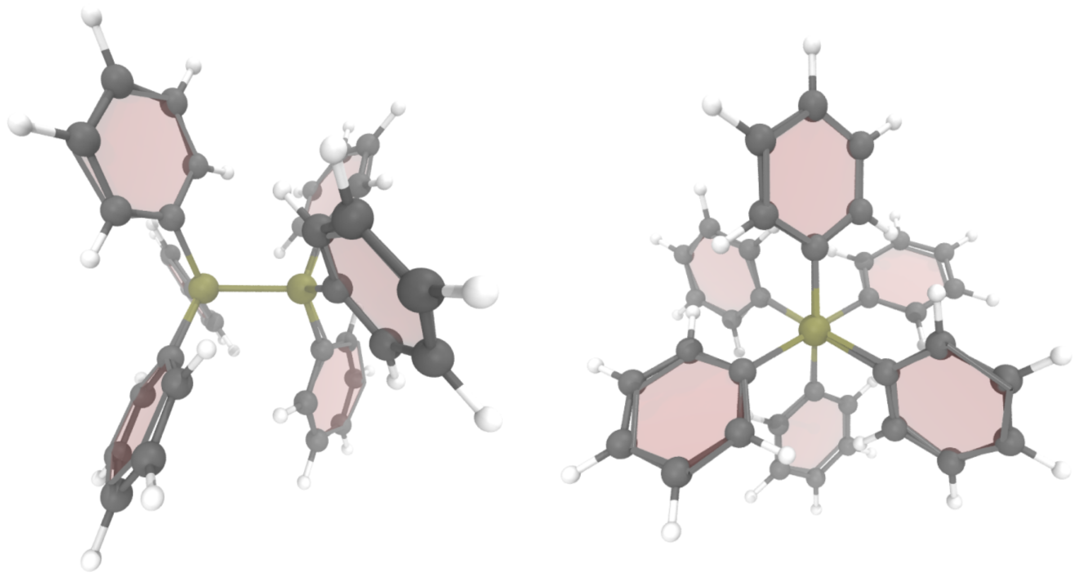

Figure S2: Overlay of the computed and crystal structure of hexaphenyldisilane.

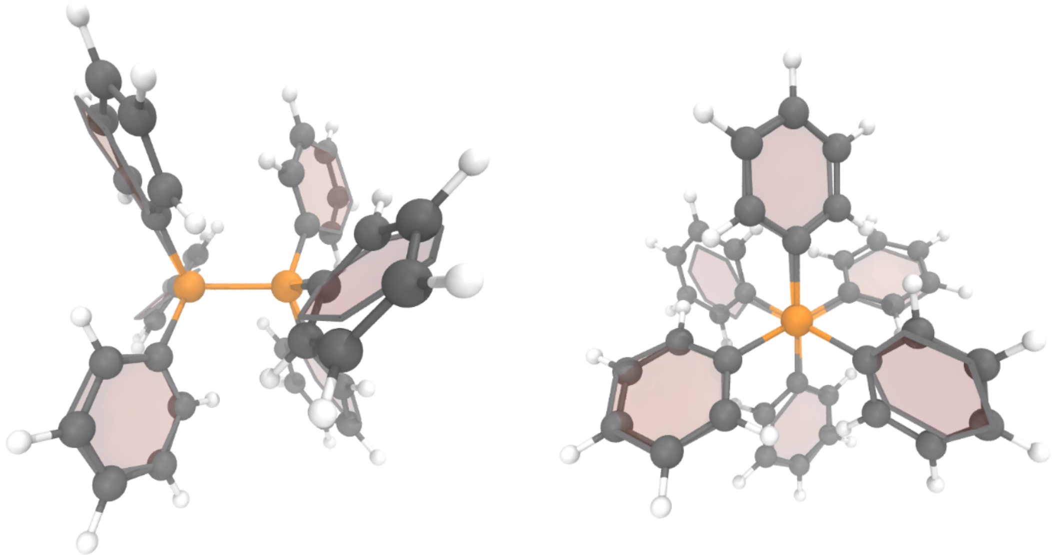

Figure S3: Overlay of the computed and crystal structure of hexaphenyldigermane.

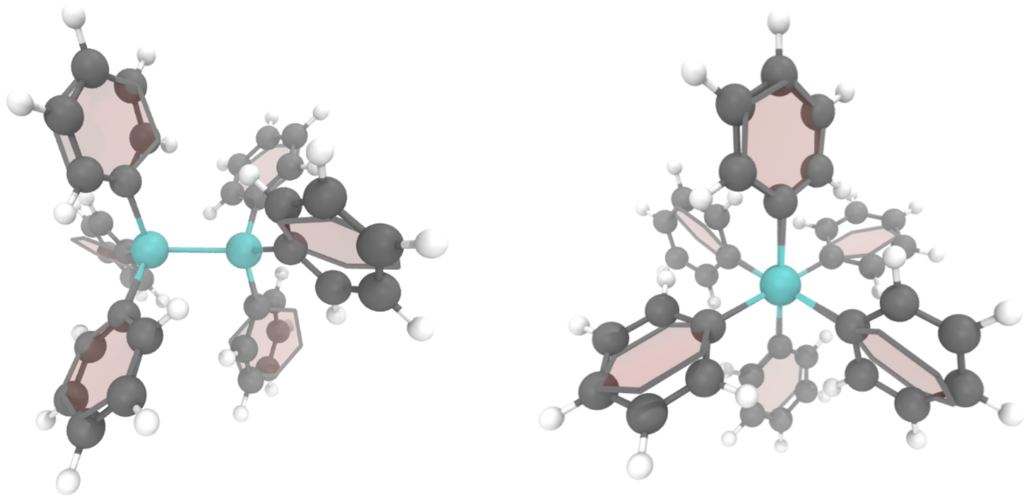

Figure S4: Overlay of the computed and crystal structure of hexaphenyldistannane.

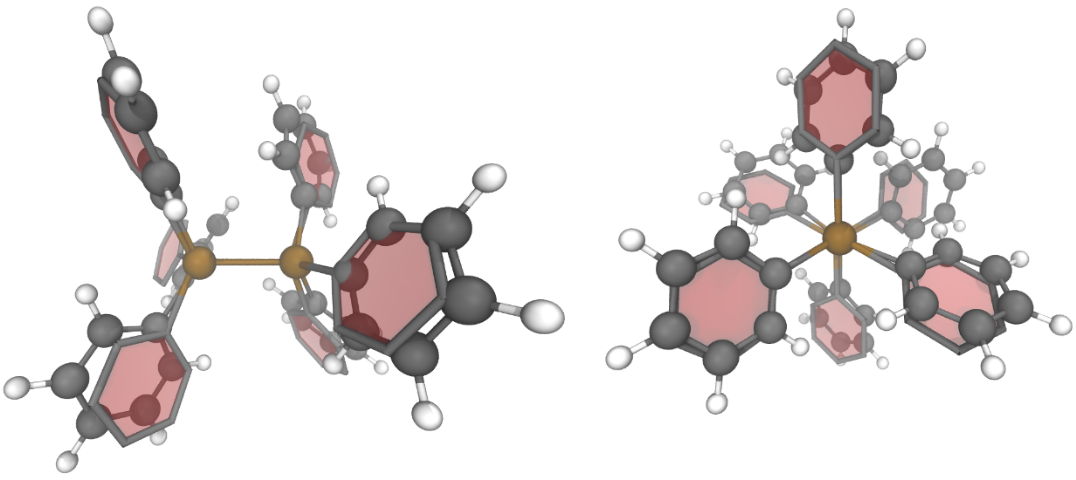

Figure S5: Overlay of the computed and crystal structure of hexaphenyldiplumbane.

Table S10: Comparison of experimental and computational distances *d*(T–T), *d*(CH-π) and point groups

T	*d*(T–T) [Å]	exp *d*(T–T) [Å][a]	*d*(CH-π) [Å]	exp *d*(CH-π) [Å]	point group
C	1.702	n.a.	2.498	n.a.	S_6
Si	2.350	2.462	3.043	3.266	S_6
Ge	2.435	2.437	3.182	3.626	S_6
Sn	2.763	2.791	3.677	3.874<br>3.886<br>3.918	S_6
Pb	2.871	2.830	3.908	3.718<br>4.210<br>4.264	S_6^*
Pb	2.883	2.830	3.109<br>4.792<br>4.802	3.718<br>4.210<br>4.264	C_1
C–Si	1.979	n.a.	2.893$^{(C)\ CH-\pi\ (Si)}$<br>2.627$^{(Si)\ CH-\pi\ (C)}$	n.a.	C_3

*Three imaginary frequencies below –5 cm^{-1} detected.

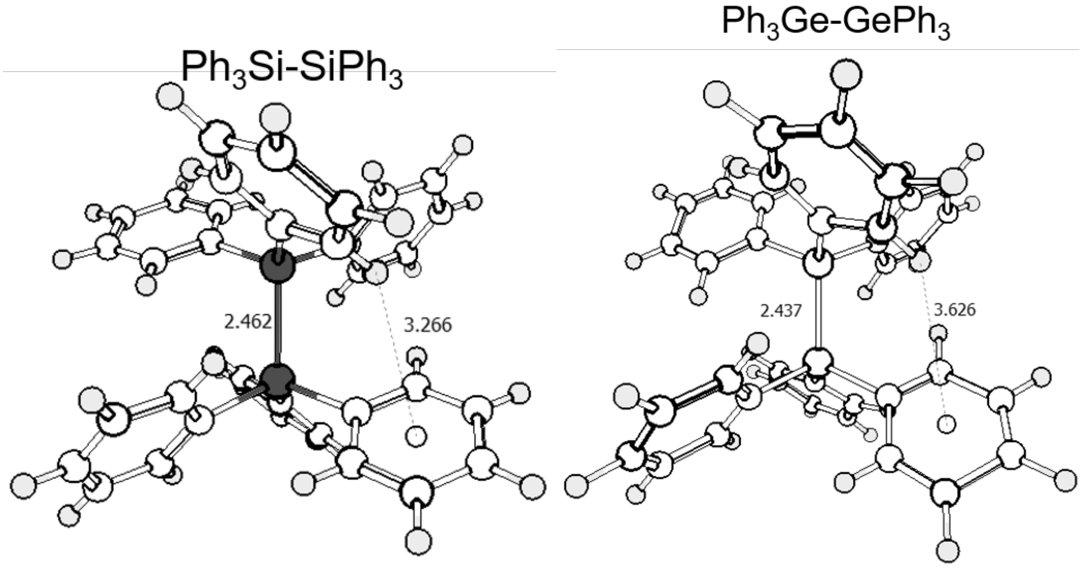

Figure S6: X-Ray Distances of Hexaphenylsilane[18] and Hexaphenylgermane[19]

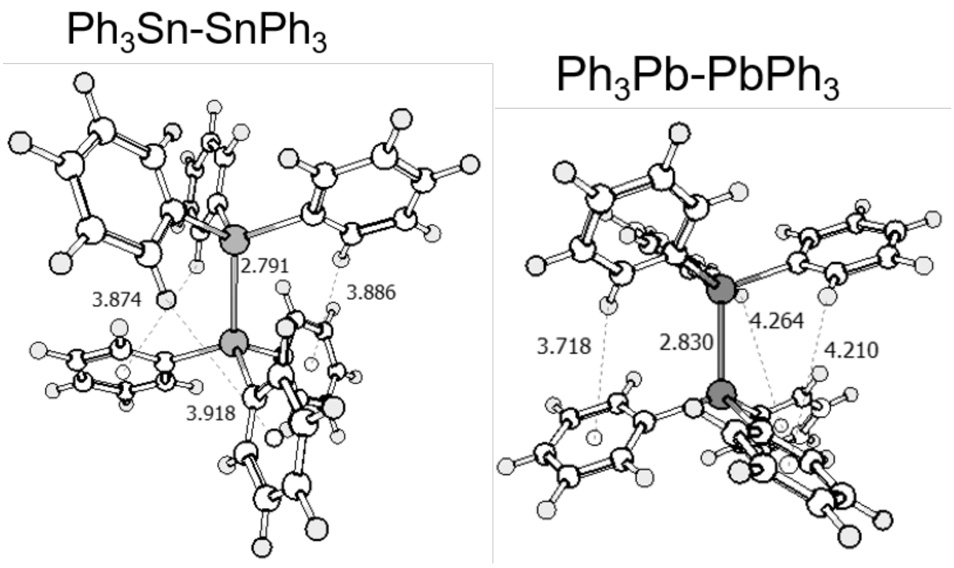

Figure S7: X-Ray Distances of Hexaphenylstannane[20, 23] and Hexaphenylplumbane[21]

3. Local Energy Decomposition (LED) analysis

In order to dissect the non-covalent interactions between the two monomer structures in the dimer geometry the two components were defined as follows:

Scheme S2: LED Definition of Fragments

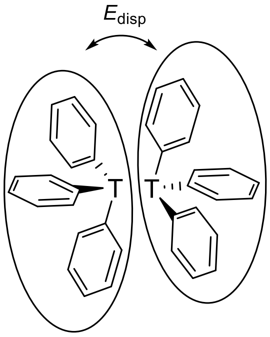

Since this splits a covalent bond homolytically, the results have to be treated with caution. Nevertheless, the dispersion interaction term can be utilized to qualitatively assess LD.

Table S4: Summary of LED computations

	E_{elst} [kcal mol^{-1}]	E_{exch} [kcal mol^{-1}]	$E_{disp(SP)}$ [kcal mol^{-1}]	$E_{CT(1-2)}$ [kcal mol^{-1}]	$E_{CT(2-1)}$ [kcal mol^{-1}]	$E_{disp(WP)}$ [kcal mol^{-1}]	E_{trip} [kcal mol^{-1}]	E_{disp} [kcal mol^{-1}]
**C–C**	−1291.8	−125.5	−37.3	−32.8	−69.1	−4.3	−13.0	**−41.7**
**Si–Si**	−1114.4	−83.8	−17.3	−42.4	−14.4	−4.0	−7.4	**−21.2**
**Ge–Ge**	−1395.1	−104.5	−16.8	−37.8	−7.5	−4.5	−7.2	**−21.3**
**Sn–Sn**	−888.5	−88.9	−11.9	−4.4	−38.2	−4.6	−5.8	**−16.5**
**Pb–Pb**	−936.9	−114.8	−14.0	−49.1	−10.1	−4.2	−7.1	**−18.2**
**C–Si**	−1039.1	−80.3	−21.7	−63.3	−16.1	−4.1	−8.5	**−25.8**

4. SAPT analysis

In order to isolate the interaction between two phenyl moieties, all other atoms were removed and both phenyl radicals were terminated with a hydrogen radical.

Scheme S3: SAPT analysis of benzyl dimers in close proximity

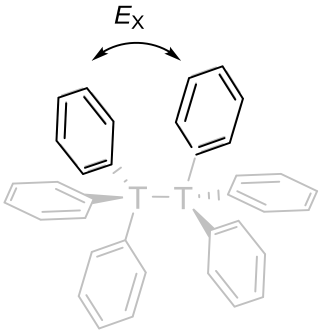

Table S5: Summary of SAPT computations

syn–*syn* R	E_{elst} [kcal mol^{-1}]	E_{ex} [kcal mol^{-1}]	E_{ind} [kcal mol^{-1}]	E_{disp} [kcal mol^{-1}]	E_{tot} [kcal mol^{-1}]
**C–C**	−6.3	17.7	−1.7	−8.3	1.5
**Si–Si**	−1.4	2.9	−0.4	−3.2	−2.1
**Ge–Ge**	−1.0	1.9	−0.3	−2.6	−2.0
**Sn–Sn**	−0.5	0.5	−0.1	−1.3	−1.4
**Pb–Pb**	−0.3	0.3	−0.1	−1.0	−1.1
**C–Si**	−3.0	7.6	−0.9	−5.8	−2.0

5. Discussion of LD assessments

In this manuscript we used three different types of computations to demonstrate the importance of LD within hexaphenylditetrels. First, we demonstrate through homodesmotic equations with and without dispersion corrected DFT methods the relative stability of the hexaphenylditetrels towards their unsubstituted precursors and found that dispersion (–14.2 kcal mol^{-1}) counterbalances repulsion (–15.2 kcal mol^{-1}) almost perfectly **1CSi**. In all higher tetrels LD favors the hexaphenylditetrel structures (Figure 5).

The second method used is the Local Energy Decomposition (LED) analysis included in Orca. This method probably delivers a more accurate estimate of the LD energy for the two Ph_3T fragments. It demonstrates a clear trend from –42 kcal mol^{-1} LD energy in **1C** to –18 kcal mol^{-1} in **1Pb**. However, the repulsive term of the Lennard-Jones potential cannot be estimated well with this method. These numbers are reasonable since the dimers are large and include heavy tetrel contacts (Figure 4).

Furthermore, we investigated the energetic contribution of each “off-shape” phenyl contact within the described hexaphenylditetrels with density fitted symmetry adapted perturbation theory and estimate LD to amount to about –8 kcal mol^{-1} in **1C** per CH-π contact while the higher congeners benefit ~–1 to –3 kcal mol^{-1} from LD interactions. However, the close distances between the phenyl groups in **1C** results in stronger repulsion (~+18 kcal mol^{-1}) that counterbalances LD, thereby destabilizing **1C** (Figure 6).

All methods demonstrate that **1C** is too labile to be isolable and that it is a clear outlier within the tetrel series. Even though the LD contribution is the largest, the short T-T bond in **1C** results in close phenyl contacts and large repulsive interactions. Consequently, longer bonds provide higher stability within the hexaphenylditetrel series.

6. Cartesian Coordinates

6.1. Ph_3T-TPh_3 Structures

Hexaphenylethane – B3LYP/def2-TZVP

6	0.000000000	0.000000000	0.865610000
6	0.000000000	0.000000000	-0.865610000
6	0.402180000	-1.401714000	1.437772000
6	-0.371898000	-2.051607000	2.404803000
6	0.000000000	-3.278539000	2.949436000
6	1.172916000	-3.900367000	2.553704000
6	1.980923000	-3.256817000	1.623834000
6	1.608683000	-2.030653000	1.090498000
1	-1.288244000	-1.608742000	2.757673000
1	-0.640539000	-3.740613000	3.690572000
1	1.462018000	-4.856891000	2.970597000
6	1.012830000	1.049155000	1.437772000
6	1.962693000	0.703730000	2.404803000
6	0.954256000	2.408487000	1.090498000
6	1.830024000	3.343938000	1.623834000
6	2.839298000	1.639270000	2.949436000
6	2.791359000	2.965959000	2.553704000
1	3.475182000	3.694590000	2.970597000
1	1.748723000	4.378069000	1.312338000
1	2.037333000	-0.311281000	2.757673000
1	3.559735000	1.315584000	3.690572000
1	0.210411000	2.760454000	0.398261000
6	-1.415009000	0.352559000	1.437772000
6	-2.562939000	-0.377834000	1.090498000
6	-1.590795000	1.347877000	2.404803000
6	-2.839298000	1.639270000	2.949436000
6	-3.810947000	-0.087121000	1.623834000
6	-3.964275000	0.934408000	2.553704000
1	-2.495828000	-1.198006000	0.398261000
1	-4.665881000	-0.674596000	1.312338000
1	-0.749089000	1.920023000	2.757673000
1	-4.937200000	1.162301000	2.970597000
1	-2.919196000	2.425029000	3.690572000
6	1.415009000	-0.352559000	-1.437772000
6	2.562939000	0.377834000	-1.090498000
6	3.810947000	0.087121000	-1.623834000
6	3.964275000	-0.934408000	-2.553704000
6	2.839298000	-1.639270000	-2.949436000
6	1.590795000	-1.347877000	-2.404803000
1	0.749089000	-1.920023000	-2.757673000
1	4.937200000	-1.162301000	-2.970597000
1	2.919196000	-2.425029000	-3.690572000
1	2.495828000	1.198006000	-0.398261000
1	4.665881000	0.674596000	-1.312338000
6	-1.012830000	-1.049155000	-1.437772000
6	-0.954256000	-2.408487000	-1.090498000
6	-1.830024000	-3.343938000	-1.623834000
6	-2.791359000	-2.965959000	-2.553704000
6	-2.839298000	-1.639270000	-2.949436000
6	-1.962693000	-0.703730000	-2.404803000
1	-0.210411000	-2.760454000	-0.398261000
1	-1.748723000	-4.378069000	-1.312338000
1	-3.475182000	-3.694590000	-2.970597000
1	-3.559735000	-1.315584000	-3.690572000
1	-2.037333000	0.311281000	-2.757673000
6	-0.402180000	1.401714000	-1.437772000
6	-1.608683000	2.030653000	-1.090498000
6	0.371898000	2.051607000	-2.404803000
6	0.000000000	3.278539000	-2.949436000
6	-1.980923000	3.256817000	-1.623834000
6	-1.172916000	3.900367000	-2.553704000
1	-2.917157000	3.703473000	-1.312338000
1	-1.462018000	4.856891000	-2.970597000
1	-2.285418000	1.562448000	-0.398261000
1	1.288244000	1.608742000	-2.757673000
1	0.640539000	3.740613000	-3.690572000
1	2.285418000	-1.562448000	0.398261000
1	2.917157000	-3.703473000	1.312338000

E = -1466.55716485 au

ZPVE = 0.560866 au

Hexaphenylethane – B3LYP-D3(BJ)/def2-TZVP

6	0.000000000	0.000000000	0.850910000
6	0.000000000	0.000000000	-0.850910000
6	0.417790000	-1.391102000	1.405156000
6	-0.367795000	-2.076884000	2.332739000
6	0.000000000	-3.323810000	2.829820000
6	1.181232000	-3.923035000	2.425174000
6	2.006549000	-3.234241000	1.544121000
6	1.638252000	-1.989334000	1.058572000
1	-1.291466000	-1.649354000	2.683555000
1	-0.650041000	-3.820838000	3.538661000
1	1.465656000	-4.896966000	2.801980000
6	0.995835000	1.057368000	1.405156000
6	1.982531000	0.719922000	2.332739000
6	0.903688000	2.413435000	1.058572000
6	1.797660000	3.354843000	1.544121000
6	2.878504000	1.661905000	2.829820000
6	2.806832000	2.984494000	2.425174000
1	3.508069000	3.717778000	2.801980000
1	1.695293000	4.386699000	1.233128000
1	2.074116000	-0.293765000	2.683555000
1	3.633963000	1.347467000	3.538661000
1	0.123883000	2.755003000	0.403690000
6	-1.413625000	0.333735000	1.405156000
6	-2.541940000	-0.424101000	1.058572000
6	-1.614737000	1.356961000	2.332739000
6	-2.878504000	1.661905000	2.829820000
6	-3.804209000	-0.120602000	1.544121000
6	-3.988064000	0.938541000	2.425174000
1	-2.447844000	-1.270216000	0.403690000
1	-4.646639000	-0.725183000	1.233128000
1	-0.782650000	1.943120000	2.683555000
1	-4.973725000	1.179188000	2.801980000
1	-2.983922000	2.473371000	3.538661000
6	1.413625000	-0.333735000	-1.405156000
6	2.541940000	0.424101000	-1.058572000
6	3.804209000	0.120602000	-1.544121000
6	3.988064000	-0.938541000	-2.425174000
6	2.878504000	-1.661905000	-2.829820000
6	1.614737000	-1.356961000	-2.332739000
1	0.782650000	-1.943120000	-2.683555000
1	4.973725000	-1.179188000	-2.801980000
1	2.983922000	-2.473371000	-3.538661000
1	2.447844000	1.270216000	-0.403690000
1	4.646639000	0.725183000	-1.233128000
6	-0.995835000	-1.057368000	-1.405156000
6	-0.903688000	-2.413435000	-1.058572000
6	-1.797660000	-3.354843000	-1.544121000
6	-2.806832000	-2.984494000	-2.425174000
6	-2.878504000	-1.661905000	-2.829820000
6	-1.982531000	-0.719922000	-2.332739000
1	-0.123883000	-2.755003000	-0.403690000
1	-1.695293000	-4.386699000	-1.233128000

1	-3.508069000	-3.717778000	-2.801980000
1	-3.633963000	-1.347467000	-3.538661000
1	-2.074116000	0.293765000	-2.683555000
6	-0.417790000	1.391102000	-1.405156000
6	-1.638252000	1.989334000	-1.058572000
6	0.367795000	2.076884000	-2.332739000
6	0.000000000	3.323810000	-2.829820000
6	-2.006549000	3.234241000	-1.544121000
6	-1.181232000	3.923035000	-2.425174000
1	-2.951346000	3.661516000	-1.233128000
1	-1.465656000	4.896966000	-2.801980000
1	-2.323962000	1.484787000	-0.403690000
1	1.291466000	1.649354000	-2.683555000
1	0.650041000	3.820838000	-3.538661000
1	2.323962000	-1.484787000	0.403690000
1	2.951346000	-3.661516000	1.233128000

E = -1466.76853225 au

ZPVE = 0.562865 au

Hexaphenylethane – M06-2X/cc-pVDZ/SDD

6	0.000000000	0.000000000	0.846011000
6	0.000000000	0.000000000	-0.846011000
6	0.426116000	-1.392461000	1.401184000
6	-0.368124000	-2.102073000	2.311264000
6	0.000000000	-3.364249000	2.785778000
6	1.189709000	-3.954331000	2.380275000
6	2.029861000	-3.237677000	1.527832000
6	1.662827000	-1.979333000	1.062383000
1	-1.302019000	-1.681916000	2.673800000
1	-0.661726000	-3.880755000	3.482434000
1	1.473434000	-4.944046000	2.740078000
6	0.992848000	1.065258000	1.401184000
6	2.004511000	0.732232000	2.311264000
6	0.882740000	2.429717000	1.062383000
6	1.788980000	3.376749000	1.527832000
6	2.913525000	1.682125000	2.785778000
6	2.829697000	3.007483000	2.380275000
1	3.544953000	3.748054000	2.740078000
1	1.669439000	4.417227000	1.222614000
1	2.107591000	-0.286624000	2.673800000
1	3.691695000	1.367305000	3.482434000
1	0.070015000	2.779676000	0.433825000
6	-1.418965000	0.327203000	1.401184000
6	-2.545566000	-0.450384000	1.062383000
6	-1.636387000	1.369841000	2.311264000
6	-2.913525000	1.682125000	2.785778000
6	-3.818840000	-0.139073000	1.527832000
6	-4.019405000	0.946848000	2.380275000
1	-2.442278000	-1.329203000	0.433825000
1	-4.660151000	-0.762837000	1.222614000
1	-0.805572000	1.968540000	2.673800000
1	-5.018387000	1.195992000	2.740078000
1	-3.029969000	2.513449000	3.482434000
6	1.418965000	-0.327203000	-1.401184000
6	2.545566000	0.450384000	-1.062383000
6	3.818840000	0.139073000	-1.527832000
6	4.019405000	-0.946848000	-2.380275000
6	2.913525000	-1.682125000	-2.785778000
6	1.636387000	-1.369841000	-2.311264000
1	0.805572000	-1.968540000	-2.673800000
1	5.018387000	-1.195992000	-2.740078000
1	3.029969000	-2.513449000	-3.482434000
1	2.442278000	1.329203000	-0.433825000
1	4.660151000	0.762837000	-1.222614000
6	-0.992848000	-1.065258000	-1.401184000
6	-0.882740000	-2.429717000	-1.062383000
6	-1.788980000	-3.376749000	-1.527832000
6	-2.829697000	-3.007483000	-2.380275000
6	-2.913525000	-1.682125000	-2.785778000
6	-2.004511000	-0.732232000	-2.311264000
1	-0.070015000	-2.779676000	-0.433825000
1	-1.669439000	-4.417227000	-1.222614000
1	-3.544953000	-3.748054000	-2.740078000
1	-3.691695000	-1.367305000	-3.482434000
1	-2.107591000	0.286624000	-2.673800000
6	-0.426116000	1.392461000	-1.401184000
6	-1.662827000	1.979333000	-1.062383000
6	0.368124000	2.102073000	-2.311264000
6	0.000000000	3.364249000	-2.785778000
6	-2.029861000	3.237677000	-1.527832000
6	-1.189709000	3.954331000	-2.380275000
1	-2.990711000	3.654390000	-1.222614000
1	-1.473434000	4.944046000	-2.740078000
1	-2.372263000	1.450473000	-0.433825000
1	1.302019000	1.681916000	-2.673800000
1	0.661726000	3.880755000	-3.482434000
1	2.372263000	-1.450473000	0.433825000
1	2.990711000	-3.654390000	1.222614000

E = -1465.61445392 au

ZPVE = 0.566687 au

Hexaphenyldisilane – B3LYP/def2-TZVP

14	0.000000000	0.000000000	1.201811000
14	0.000000000	0.000000000	-1.201811000
6	-1.076947000	1.418483000	1.852855000
6	-0.883777000	2.734869000	1.406120000
6	-1.656022000	3.784014000	1.890549000
6	-2.646243000	3.542228000	2.837111000
6	-2.854992000	2.247066000	3.294779000
6	-2.078607000	1.200084000	2.807407000
1	-0.121056000	2.949687000	0.667190000
1	-1.485289000	4.790128000	1.527334000
1	-3.250125000	4.358247000	3.214596000
1	-3.622573000	2.049044000	4.033118000
1	-2.258609000	0.198722000	3.177927000
6	1.766916000	0.223422000	1.852855000
6	-0.689969000	-1.641905000	1.852855000
6	-1.926578000	-2.132807000	1.406120000
6	0.000000000	-2.400168000	2.807407000
6	-2.449041000	-3.326165000	1.890549000
6	-1.744538000	-4.062828000	2.837111000
6	-0.518521000	-3.596029000	3.294779000
1	0.957206000	-2.055374000	3.177927000
1	-2.493976000	-1.579682000	0.667190000
1	-3.405728000	-3.681362000	1.527334000
1	-2.149290000	-4.993815000	3.214596000
1	0.036762000	-4.161762000	4.033118000
6	2.810354000	-0.602061000	1.406120000
6	2.078607000	1.200084000	2.807407000
6	3.373512000	1.348962000	3.294779000
6	4.390781000	0.520600000	2.837111000
6	4.105064000	-0.457850000	1.890549000
1	2.615032000	-1.370006000	0.667190000
1	1.301403000	1.856651000	3.177927000
1	3.585810000	2.112718000	4.033118000
1	5.399415000	0.635567000	3.214596000
1	4.891017000	-1.108766000	1.527334000
6	0.689969000	1.641905000	-1.852855000
6	-1.766916000	-0.223422000	-1.852855000
6	1.076947000	-1.418483000	-1.852855000
6	2.078607000	-1.200084000	-2.807407000
6	2.854992000	-2.247066000	-3.294779000
6	0.883777000	-2.734869000	-1.406120000
6	1.656022000	-3.784014000	-1.890549000
6	2.646243000	-3.542228000	-2.837111000
1	2.258609000	-0.198722000	-3.177927000
1	3.250125000	-4.358247000	-3.214596000
1	3.622573000	-2.049044000	-4.033118000

1	0.121056000	-2.949687000	-0.667190000
1	1.485289000	-4.790128000	-1.527334000
6	0.000000000	2.400168000	-2.807407000
6	1.926578000	2.132807000	-1.406120000
6	2.449041000	3.326165000	-1.890549000
6	1.744538000	4.062828000	-2.837111000
6	0.518521000	3.596029000	-3.294779000
1	-0.036762000	4.161762000	-4.033118000
1	-0.957206000	2.055374000	-3.177927000
1	2.493976000	1.579682000	-0.667190000
1	3.405728000	3.681362000	-1.527334000
1	2.149290000	4.993815000	-3.214596000
6	-2.810354000	0.602061000	-1.406120000
6	-2.078607000	-1.200084000	-2.807407000
6	-3.373512000	-1.348962000	-3.294779000
6	-4.105064000	0.457850000	-1.890549000
6	-4.390781000	-0.520600000	-2.837111000
1	-5.399415000	-0.635567000	-3.214596000
1	-3.585810000	-2.112718000	-4.033118000
1	-2.615032000	1.370006000	-0.667190000
1	-4.891017000	1.108766000	-1.527334000
1	-1.301403000	-1.856651000	-3.177927000

E = -1969.49238404 au

ZPVE = 0.546851 au

Hexaphenyldisilane – B3LYP-D3(BJ)/def2-TZVP

14	0.000000000	0.000000000	1.174846000
14	0.000000000	0.000000000	-1.174846000
6	-1.056808000	1.436439000	1.762039000
6	-0.786667000	2.737035000	1.313237000
6	-1.591279000	3.805581000	1.685048000
6	-2.688318000	3.595830000	2.514452000
6	-2.969981000	2.314689000	2.972367000
6	-2.160508000	1.247370000	2.599083000
1	0.054113000	2.917476000	0.655405000
1	-1.365743000	4.800857000	1.323231000
1	-3.319463000	4.427311000	2.801643000
1	-3.821188000	2.145039000	3.619886000
1	-2.395689000	0.253811000	2.958852000
6	1.772397000	0.197003000	1.762039000
6	-0.715589000	-1.633442000	1.762039000
6	-1.977009000	-2.049791000	1.313237000
6	0.000000000	-2.494740000	2.599083000
6	-2.500091000	-3.280878000	1.685048000
6	-1.769921000	-4.126066000	2.514452000
6	-0.519589000	-3.729424000	2.972367000
1	0.978037000	-2.201633000	2.958852000
1	-2.553665000	-1.411875000	0.655405000
1	-3.474793000	-3.583197000	1.323231000
1	-2.174432000	-5.088394000	2.801643000
1	0.052936000	-4.381766000	3.619886000
6	2.763676000	-0.687244000	1.313237000
6	2.160508000	1.247370000	2.599083000
6	3.489570000	1.414735000	2.972367000
6	4.458239000	0.530236000	2.514452000
6	4.091369000	-0.524703000	1.685048000
1	2.499552000	-1.505601000	0.655405000
1	1.417652000	1.947822000	2.958852000
1	3.768252000	2.236727000	3.619886000
1	5.493895000	0.661084000	2.801643000
1	4.840536000	-1.217661000	1.323231000
6	0.715589000	1.633442000	-1.762039000
6	-1.772397000	-0.197003000	-1.762039000
6	1.056808000	-1.436439000	-1.762039000
6	2.160508000	-1.247370000	-2.599083000
6	2.969981000	-2.314689000	-2.972367000
6	0.786667000	-2.737035000	-1.313237000
6	1.591279000	-3.805581000	-1.685048000
6	2.688318000	-3.595830000	-2.514452000
1	2.395689000	-0.253811000	-2.958852000
1	3.319463000	-4.427311000	-2.801643000
1	3.821188000	-2.145039000	-3.619886000
1	-0.054113000	-2.917476000	-0.655405000
1	1.365743000	-4.800857000	-1.323231000
6	0.000000000	2.494740000	-2.599083000
6	1.977009000	2.049791000	-1.313237000
6	2.500091000	3.280878000	-1.685048000
6	1.769921000	4.126066000	-2.514452000
6	0.519589000	3.729424000	-2.972367000
1	-0.052936000	4.381766000	-3.619886000
1	-0.978037000	2.201633000	-2.958852000
1	2.553665000	1.411875000	-0.655405000
1	3.474793000	3.583197000	-1.323231000
1	2.174432000	5.088394000	-2.801643000
6	-2.763676000	0.687244000	-1.313237000
6	-2.160508000	-1.247370000	-2.599083000
6	-3.489570000	-1.414735000	-2.972367000
6	-4.091369000	0.524703000	-1.685048000
6	-4.458239000	-0.530236000	-2.514452000
1	-5.493895000	-0.661084000	-2.801643000
1	-3.768252000	-2.236727000	-3.619886000
1	-2.499552000	1.505601000	-0.655405000
1	-4.840536000	1.217661000	-1.323231000
1	-1.417652000	-1.947822000	-2.958852000

E = -1969.69167141 au

ZPVE = 0.548318 au

Hexaphenyldisilane – M06-2X/ cc-pVDZ/SDD

14	0.000000000	0.000000000	1.178840000
14	0.000000000	0.000000000	-1.178840000
6	0.729619000	1.645275000	1.760687000
6	2.007199000	2.039528000	1.320879000
6	2.532404000	3.283435000	1.666224000
6	1.787629000	4.161188000	2.455475000
6	0.522570000	3.785700000	2.903335000
6	0.000000000	2.538159000	2.558865000
1	2.599978000	1.369049000	0.691063000
1	3.523967000	3.570354000	1.312941000
1	2.195400000	5.137542000	2.721750000
1	-0.062066000	4.466705000	3.523805000
1	-0.994465000	2.257548000	2.913738000
6	1.060041000	-1.454506000	1.760687000
6	-1.789660000	-0.190769000	1.760687000
6	-2.769882000	0.718522000	1.320879000
6	-2.198110000	-1.269079000	2.558865000
6	-4.109740000	0.551409000	1.666224000
6	-4.497509000	-0.532462000	2.455475000
6	-3.539797000	-1.440291000	2.903335000
1	-1.457862000	-1.990005000	2.913738000
1	-2.485620000	1.567123000	0.691063000
1	-4.854001000	1.266668000	1.312941000
1	-5.546942000	-0.667499000	2.721750000
1	-3.837247000	-2.287103000	3.523805000
6	0.762683000	-2.758049000	1.320879000
6	2.198110000	-1.269079000	2.558865000
6	3.017227000	-2.345409000	2.903335000
6	2.709881000	-3.628726000	2.455475000
6	1.577336000	-3.834844000	1.666224000
1	-0.114358000	-2.936172000	0.691063000
1	2.452326000	-0.267542000	2.913738000
1	3.899313000	-2.179602000	3.523805000
1	3.351542000	-4.470043000	2.721750000
1	1.330034000	-4.837022000	1.312941000
6	1.789660000	0.190769000	-1.760687000
6	-1.060041000	1.454506000	-1.760687000
6	-0.729619000	-1.645275000	-1.760687000
6	0.000000000	-2.538159000	-2.558865000

6	-0.522570000	-3.785700000	-2.903335000
6	-2.007199000	-2.039528000	-1.320879000
6	-2.532404000	-3.283435000	-1.666224000
6	-1.787629000	-4.161188000	-2.455475000
1	0.994465000	-2.257548000	-2.913738000
1	-2.195400000	-5.137542000	-2.721750000
1	0.062066000	-4.466705000	-3.523805000
1	-2.599978000	-1.369049000	-0.691063000
1	-3.523967000	-3.570354000	-1.312941000
6	2.198110000	1.269079000	-2.558865000
6	2.769882000	-0.718522000	-1.320879000
6	4.109740000	-0.551409000	-1.666224000
6	4.497509000	0.532462000	-2.455475000
6	3.539797000	1.440291000	-2.903335000
1	3.837247000	2.287103000	-3.523805000
1	1.457862000	1.990005000	-2.913738000
1	2.485620000	-1.567123000	-0.691063000
1	4.854001000	-1.266668000	-1.312941000
1	5.546942000	0.667499000	-2.721750000
6	-0.762683000	2.758049000	-1.320879000
6	-2.198110000	1.269079000	-2.558865000
6	-3.017227000	2.345409000	-2.903335000
6	-1.577336000	3.834844000	-1.666224000
6	-2.709881000	3.628726000	-2.455475000
1	-3.351542000	4.470043000	-2.721750000
1	-3.899313000	2.179602000	-3.523805000
1	0.114358000	2.936172000	-0.691063000
1	-1.330034000	4.837022000	-1.312941000
1	-2.452326000	0.267542000	-2.913738000

E = -1968.46043334 au

ZPVE = 0.551496 au

Hexaphenyldigermane – B3LYP/def2-TZVP

32	0.000000000	0.000000000	1.245587000
32	0.000000000	0.000000000	-1.245587000
6	-1.138776000	1.465263000	1.935259000
6	-0.977193000	2.776524000	1.473016000
6	-1.766684000	3.810948000	1.964083000
6	-2.735626000	3.551922000	2.927706000
6	-2.908769000	2.255187000	3.396669000
6	-2.116704000	1.222079000	2.903726000
1	-0.230842000	3.001165000	0.720031000
1	-1.625271000	4.818591000	1.592513000
1	-3.351859000	4.356345000	3.309938000
1	-3.660827000	2.045081000	4.147576000
1	-2.266714000	0.217690000	3.280496000
6	1.838343000	0.253578000	1.935259000
6	-0.699567000	-1.718841000	1.935259000
6	-1.915944000	-2.234535000	1.473016000
6	0.000000000	-2.444159000	2.903726000
6	-2.417036000	-3.435467000	1.964083000
6	-1.708242000	-4.145083000	2.927706000
6	-0.498664000	-3.646661000	3.396669000
1	0.944832000	-2.071877000	3.280496000
1	-2.483664000	-1.700498000	0.720031000
1	-3.360386000	-3.816822000	1.592513000
1	-2.096776000	-5.080968000	3.309938000
1	0.059321000	-4.192910000	4.147576000
6	2.893136000	-0.541988000	1.473016000
6	2.116704000	1.222079000	2.903726000
6	3.407433000	1.391474000	3.396669000
6	4.443868000	0.593161000	2.927706000
6	4.183719000	-0.375481000	1.964083000
1	2.714506000	-1.300667000	0.720031000
1	1.321882000	1.854187000	3.280496000
1	3.601506000	2.147828000	4.147576000
1	5.448635000	0.724623000	3.309938000
1	4.985658000	-1.001769000	1.592513000
6	0.699567000	1.718841000	-1.935259000
6	-1.838343000	-0.253578000	-1.935259000
6	1.138776000	-1.465263000	-1.935259000
6	2.116704000	-1.222079000	-2.903726000
6	2.908769000	-2.255187000	-3.396669000
6	0.977193000	-2.776524000	-1.473016000
6	1.766684000	-3.810948000	-1.964083000
6	2.735626000	-3.551922000	-2.927706000
1	2.266714000	-0.217690000	-3.280496000
1	3.351859000	-4.356345000	-3.309938000
1	3.660827000	-2.045081000	-4.147576000
1	0.230842000	-3.001165000	-0.720031000
1	1.625271000	-4.818591000	-1.592513000
6	0.000000000	2.444159000	-2.903726000
6	1.915944000	2.234535000	-1.473016000
6	2.417036000	3.435467000	-1.964083000
6	1.708242000	4.145083000	-2.927706000
6	0.498664000	3.646661000	-3.396669000
1	-0.059321000	4.192910000	-4.147576000
1	-0.944832000	2.071877000	-3.280496000
1	2.483664000	1.700498000	-0.720031000
1	3.360386000	3.816822000	-1.592513000
1	2.096776000	5.080968000	-3.309938000
6	-2.893136000	0.541988000	-1.473016000
6	-2.116704000	-1.222079000	-2.903726000
6	-3.407433000	-1.391474000	-3.396669000
6	-4.183719000	0.375481000	-1.964083000
6	-4.443868000	-0.593161000	-2.927706000
1	-5.448635000	-0.724623000	-3.309938000
1	-3.601506000	-2.147828000	-4.147576000
1	-2.714506000	1.300667000	-0.720031000
1	-4.985658000	1.001769000	-1.592513000
1	-1.321882000	-1.854187000	-3.280496000

E = -5544.53328022 au

ZPVE = 0.543291 au

Hexaphenyldigermane – B3LYP-D3(BJ) /def2-TZVP

32	0.000000000	0.000000000	1.217579000
32	0.000000000	0.000000000	-1.217579000
6	-1.121630000	1.482625000	1.836622000
6	-0.885997000	2.778050000	1.365485000
6	-1.705692000	3.832470000	1.748662000
6	-2.776411000	3.605851000	2.607210000
6	-3.020685000	2.322814000	3.082068000
6	-2.197824000	1.268914000	2.698298000
1	-0.064926000	2.967825000	0.684851000
1	-1.511519000	4.829411000	1.373377000
1	-3.417892000	4.426118000	2.903414000
1	-3.853079000	2.141186000	3.750509000
1	-2.400896000	0.271978000	3.069472000
6	1.844806000	0.230048000	1.836622000
6	-0.723176000	-1.712673000	1.836622000
6	-1.962863000	-2.156321000	1.365485000
6	0.000000000	-2.537829000	2.698298000
6	-2.466170000	-3.393407000	1.748662000
6	-1.734553000	-4.207368000	2.607210000
6	-0.501274000	-3.777397000	3.082068000
1	0.964908000	-2.215227000	3.069472000
1	-2.537749000	-1.540140000	0.684851000
1	-3.426633000	-3.723719000	1.373377000
1	-2.124184000	-5.173040000	2.903414000
1	0.072218000	-4.407457000	3.750509000
6	2.848860000	-0.621729000	1.365485000
6	2.197824000	1.268914000	2.698298000
6	3.521959000	1.454583000	3.082068000
6	4.510964000	0.601517000	2.607210000
6	4.171862000	-0.439062000	1.748662000
1	2.602675000	-1.427685000	0.684851000

1	1.435988000	1.943248000	3.069472000
1	3.780861000	2.266271000	3.750509000
1	5.542077000	0.746923000	2.903414000
1	4.938152000	-1.105692000	1.373377000
6	0.723176000	1.712673000	-1.836622000
6	-1.844806000	-0.230048000	-1.836622000
6	1.121630000	-1.482625000	-1.836622000
6	2.197824000	-1.268914000	-2.698298000
6	3.020685000	-2.322814000	-3.082068000
6	0.885997000	-2.778050000	-1.365485000
6	1.705692000	-3.832470000	-1.748662000
6	2.776411000	-3.605851000	-2.607210000
1	2.400896000	-0.271978000	-3.069472000
1	3.417892000	-4.426118000	-2.903414000
1	3.853079000	-2.141186000	-3.750509000
1	0.064926000	-2.967825000	-0.684851000
1	1.511519000	-4.829411000	-1.373377000
6	0.000000000	2.537829000	-2.698298000
6	1.962863000	2.156321000	-1.365485000
6	2.466170000	3.393407000	-1.748662000
6	1.734553000	4.207368000	-2.607210000
6	0.501274000	3.777397000	-3.082068000
1	-0.072218000	4.407457000	-3.750509000
1	-0.964908000	2.215227000	-3.069472000
1	2.537749000	1.540140000	-0.684851000
1	3.426633000	3.723719000	-1.373377000
1	2.124184000	5.173040000	-2.903414000
6	-2.848860000	0.621729000	-1.365485000
6	-2.197824000	-1.268914000	-2.698298000
6	-3.521959000	-1.454583000	-3.082068000
6	-4.171862000	0.439062000	-1.748662000
6	-4.510964000	-0.601517000	-2.607210000
1	-5.542077000	-0.746923000	-2.903414000
1	-3.780861000	-2.266271000	-3.750509000
1	-2.602675000	1.427685000	-0.684851000
1	-4.938152000	1.105692000	-1.373377000
1	-1.435988000	-1.943248000	-3.069472000

E = -5544.72654456 au

ZPVE = 0.544459 au

Hexaphenyldigermane – M06-2X/ cc-pVDZ/SDD

32	0.000000000	0.000000000	1.222666000
32	0.000000000	0.000000000	-1.222666000
6	0.498614000	1.799620000	1.834212000
6	1.689034000	2.388196000	1.374693000
6	2.030970000	3.688376000	1.744988000
6	1.184539000	4.422813000	2.576506000
6	0.000000000	3.851983000	3.039018000
6	-0.339557000	2.549309000	2.669918000
1	2.357173000	1.829306000	0.711661000
1	2.958741000	4.130664000	1.378904000
1	1.449341000	5.441643000	2.863370000
1	-0.663997000	4.422780000	3.690084000
1	-1.272502000	2.112605000	3.035407000
6	1.309209000	-1.331622000	1.834212000
6	-1.807823000	-0.467997000	1.834212000
6	-2.912756000	0.268648000	1.374693000
6	-2.037988000	-1.568720000	2.669918000
6	-4.209712000	-0.085316000	1.744988000
6	-4.422538000	-1.185566000	2.576506000
6	-3.335915000	-1.925991000	3.039018000
1	-1.193319000	-2.158322000	3.035407000
1	-2.762812000	1.126719000	0.711661000
1	-5.056631000	0.497012000	1.378904000
1	-5.437272000	-1.465656000	2.863370000
1	-3.498242000	-2.786428000	3.690084000
6	1.223722000	-2.656844000	1.374693000
6	2.377545000	-0.980589000	2.669918000
6	3.335915000	-1.925991000	3.039018000
6	3.237999000	-3.237247000	2.576506000
6	2.178742000	-3.603059000	1.744988000
1	0.405639000	-2.956025000	0.711661000
1	2.465821000	0.045717000	3.035407000
1	4.162239000	-1.636352000	3.690084000
1	3.987931000	-3.975987000	2.863370000
1	2.097890000	-4.627677000	1.378904000
6	1.807823000	0.467997000	-1.834212000
6	-1.309209000	1.331622000	-1.834212000
6	-0.498614000	-1.799620000	-1.834212000
6	0.339557000	-2.549309000	-2.669918000
6	0.000000000	-3.851983000	-3.039018000
6	-1.689034000	-2.388196000	-1.374693000
6	-2.030970000	-3.688376000	-1.744988000
6	-1.184539000	-4.422813000	-2.576506000
1	1.272502000	-2.112605000	-3.035407000
1	-1.449341000	-5.441643000	-2.863370000
1	0.663997000	-4.422780000	-3.690084000
1	-2.357173000	-1.829306000	-0.711661000
1	-2.958741000	-4.130664000	-1.378904000
6	2.037988000	1.568720000	-2.669918000
6	2.912756000	-0.268648000	-1.374693000
6	4.209712000	0.085316000	-1.744988000
6	4.422538000	1.185566000	-2.576506000
6	3.335915000	1.925991000	-3.039018000
1	3.498242000	2.786428000	-3.690084000
1	1.193319000	2.158322000	-3.035407000
1	2.762812000	-1.126719000	-0.711661000
1	5.056631000	-0.497012000	-1.378904000
1	5.437272000	1.465656000	-2.863370000
6	-1.223722000	2.656844000	-1.374693000
6	-2.377545000	0.980589000	-2.669918000
6	-3.335915000	1.925991000	-3.039018000
6	-2.178742000	3.603059000	-1.744988000
6	-3.237999000	3.237247000	-2.576506000
1	-3.987931000	3.975987000	-2.863370000
1	-4.162239000	1.636352000	-3.690084000
1	-0.405639000	2.956025000	-0.711661000
1	-2.097890000	4.627677000	-1.378904000
1	-2.465821000	-0.045717000	-3.035407000

E = -5543.46855325 au

ZPVE = 0.547647 au

Hexaphenyldistannane – B3LYP/def2-TZVP

50	0.000000000	0.000000000	1.415724000
50	0.000000000	0.000000000	-1.415724000
6	-1.282793000	1.566197000	2.197250000
6	-1.185582000	2.878819000	1.724359000
6	-2.004555000	3.883442000	2.231143000
6	-2.938087000	3.589227000	3.218972000
6	-3.048281000	2.288561000	3.696792000
6	-2.226835000	1.285664000	3.188728000
1	-0.467911000	3.132583000	0.951963000
1	-1.913894000	4.894506000	1.852730000
1	-3.576985000	4.369804000	3.613227000
1	-3.774012000	2.052233000	4.465738000
1	-2.330138000	0.277440000	3.573135000
6	1.997762000	0.327833000	2.197250000
6	-0.714970000	-1.894029000	2.197250000
6	-1.900339000	-2.466154000	1.724359000
6	0.000000000	-2.571327000	3.188728000
6	-2.360882000	-3.677717000	2.231143000
6	-1.639319000	-4.339071000	3.218972000
6	-0.457812000	-3.784169000	3.696792000
1	0.924799000	-2.156679000	3.573135000
1	-2.478941000	-1.971514000	0.951963000
1	-3.281820000	-4.104733000	1.852730000
1	-1.995869000	-5.282662000	3.613227000
1	0.109720000	-4.294507000	4.465738000

6	3.085922000	-0.412665000	1.724359000
6	2.226835000	1.285664000	3.188728000
6	3.506093000	1.495608000	3.696792000
6	4.577406000	0.749844000	3.218972000
6	4.365437000	-0.205726000	2.231143000
1	2.946852000	-1.161069000	0.951963000
1	1.405339000	1.879238000	3.573135000
1	3.664292000	2.242274000	4.465738000
1	5.572854000	0.912858000	3.613227000
1	5.195713000	-0.789773000	1.852730000
6	0.714970000	1.894029000	-2.197250000
6	-1.997762000	-0.327833000	-2.197250000
6	1.282793000	-1.566197000	-2.197250000
6	2.226835000	-1.285664000	-3.188728000
6	3.048281000	-2.288561000	-3.696792000
6	1.185582000	-2.878819000	-1.724359000
6	2.004555000	-3.883442000	-2.231143000
6	2.938087000	-3.589227000	-3.218972000
1	2.330138000	-0.277440000	-3.573135000
1	3.576985000	-4.369804000	-3.613227000
1	3.774012000	-2.052233000	-4.465738000
1	0.467911000	-3.132583000	-0.951963000
1	1.913894000	-4.894506000	-1.852730000
6	0.000000000	2.571327000	-3.188728000
6	1.900339000	2.466154000	-1.724359000
6	2.360882000	3.677717000	-2.231143000
6	1.639319000	4.339071000	-3.218972000
6	0.457812000	3.784169000	-3.696792000
1	-0.109720000	4.294507000	-4.465738000
1	-0.924799000	2.156679000	-3.573135000
1	2.478941000	1.971514000	-0.951963000
1	3.281820000	4.104733000	-1.852730000
1	1.995869000	5.282662000	-3.613227000
6	-3.085922000	0.412665000	-1.724359000
6	-2.226835000	-1.285664000	-3.188728000
6	-3.506093000	-1.495608000	-3.696792000
6	-4.365437000	0.205726000	-2.231143000
6	-4.577406000	-0.749844000	-3.218972000
1	-5.572854000	-0.912858000	-3.613227000
1	-3.664292000	-2.242274000	-4.465738000
1	-2.946852000	1.161069000	-0.951963000
1	-5.195713000	0.789773000	-1.852730000
1	-1.405339000	-1.879238000	-3.573135000

E = -1819.14765709 au

ZPVE = 0.539465 au

Hexaphenyldistannane – B3LYP-D3(BJ) /def2-TZVP

50	0.000000000	0.000000000	1.381229000
50	0.000000000	0.000000000	-1.381229000
6	-1.293875000	1.562364000	2.091712000
6	-1.196326000	2.847202000	1.550819000
6	-2.051999000	3.860009000	1.969877000
6	-3.020004000	3.598397000	2.933503000
6	-3.129213000	2.323137000	3.475611000
6	-2.271244000	1.311303000	3.055601000
1	-0.453889000	3.066321000	0.791394000
1	-1.965281000	4.850999000	1.541918000
1	-3.688444000	4.385488000	3.258689000
1	-3.883597000	2.114966000	4.224277000
1	-2.371873000	0.319589000	3.481115000
6	1.999984000	0.339347000	2.091712000
6	-0.706109000	-1.901710000	2.091712000
6	-1.867587000	-2.459650000	1.550819000
6	0.000000000	-2.622606000	3.055601000
6	-2.316866000	-3.707088000	1.969877000
6	-1.606301000	-4.414598000	2.933503000
6	-0.447289000	-3.871547000	3.475611000
1	0.909164000	-2.213897000	3.481115000
1	-2.428567000	-1.926240000	0.791394000
1	-3.218448000	-4.127482000	1.541918000
1	-1.953722000	-5.387030000	3.258689000
1	0.110184000	-4.420777000	4.224277000
6	3.063913000	-0.387552000	1.550819000
6	2.271244000	1.311303000	3.055601000
6	3.576502000	1.548410000	3.475611000
6	4.626305000	0.816201000	2.933503000
6	4.368866000	-0.152921000	1.969877000
1	2.882456000	-1.140081000	0.791394000
1	1.462709000	1.894308000	3.481115000
1	3.773413000	2.305811000	4.224277000
1	5.642166000	1.001543000	3.258689000
1	5.183729000	-0.723516000	1.541918000
6	0.706109000	1.901710000	-2.091712000
6	-1.999984000	-0.339347000	-2.091712000
6	1.293875000	-1.562364000	-2.091712000
6	2.271244000	-1.311303000	-3.055601000
6	3.129213000	-2.323137000	-3.475611000
6	1.196326000	-2.847202000	-1.550819000
6	2.051999000	-3.860009000	-1.969877000
6	3.020004000	-3.598397000	-2.933503000
1	2.371873000	-0.319589000	-3.481115000
1	3.688444000	-4.385488000	-3.258689000
1	3.883597000	-2.114966000	-4.224277000
1	0.453889000	-3.066321000	-0.791394000
1	1.965281000	-4.850999000	-1.541918000
6	0.000000000	2.622606000	-3.055601000
6	1.867587000	2.459650000	-1.550819000
6	2.316866000	3.707088000	-1.969877000
6	1.606301000	4.414598000	-2.933503000
6	0.447289000	3.871547000	-3.475611000
1	-0.110184000	4.420777000	-4.224277000
1	-0.909164000	2.213897000	-3.481115000
1	2.428567000	1.926240000	-0.791394000
1	3.218448000	4.127482000	-1.541918000
1	1.953722000	5.387030000	-3.258689000
6	-3.063913000	0.387552000	-1.550819000
6	-2.271244000	-1.311303000	-3.055601000
6	-3.576502000	-1.548410000	-3.475611000
6	-4.368866000	0.152921000	-1.969877000
6	-4.626305000	-0.816201000	-2.933503000
1	-5.642166000	-1.001543000	-3.258689000
1	-3.773413000	-2.305811000	-4.224277000
1	-2.882456000	1.140081000	-0.791394000
1	-5.183729000	0.723516000	-1.541918000
1	-1.462709000	-1.894308000	-3.481115000

E = -1819.33273313 au

ZPVE = 0.540236 au

Hexaphenyldistannane – M06-2X/cc-pVDZ/SDD

50	0.000000000	0.000000000	1.400937000
50	0.000000000	0.000000000	-1.400937000
6	-1.274119000	1.578772000	2.114362000
6	-1.111918000	2.887621000	1.631917000
6	-1.945606000	3.918432000	2.066382000
6	-2.960119000	3.653460000	2.986077000
6	-3.138211000	2.357909000	3.469370000
6	-2.301350000	1.328685000	3.035299000
1	-0.326668000	3.115645000	0.903475000
1	-1.804707000	4.930161000	1.682682000
1	-3.614182000	4.458228000	3.325051000
1	-3.932605000	2.146868000	4.187165000
1	-2.455732000	0.317172000	3.421745000
6	2.004316000	0.314033000	2.114362000
6	-0.730197000	-1.892805000	2.114362000
6	-1.944794000	-2.406760000	1.631917000
6	0.000000000	-2.657370000	3.035299000
6	-2.420659000	-3.644160000	2.066382000

6	-1.683930000	-4.390268000	2.986077000
6	-0.472904000	-3.896725000	3.469370000
1	0.953187000	-2.285312000	3.421745000
1	-2.534894000	-1.840725000	0.903475000
1	-3.367291000	-4.028003000	1.682682000
1	-2.053848000	-5.359087000	3.325051000
1	0.107060000	-4.479170000	4.187165000
6	3.056712000	-0.480861000	1.631917000
6	2.301350000	1.328685000	3.035299000
6	3.611115000	1.538816000	3.469370000
6	4.644049000	0.736808000	2.986077000
6	4.366265000	-0.274272000	2.066382000
1	2.861562000	-1.274920000	0.903475000
1	1.502545000	1.968140000	3.421745000
1	3.825545000	2.332302000	4.187165000
1	5.668030000	0.900859000	3.325051000
1	5.171998000	-0.902158000	1.682682000
6	0.730197000	1.892805000	-2.114362000
6	-2.004316000	-0.314033000	-2.114362000
6	1.274119000	-1.578772000	-2.114362000
6	2.301350000	-1.328685000	-3.035299000
6	3.138211000	-2.357909000	-3.469370000
6	1.111918000	-2.887621000	-1.631917000
6	1.945606000	-3.918432000	-2.066382000
6	2.960119000	-3.653460000	-2.986077000
1	2.455732000	-0.317172000	-3.421745000
1	3.614182000	-4.458228000	-3.325051000
1	3.932605000	-2.146868000	-4.187165000
1	0.326668000	-3.115645000	-0.903475000
1	1.804707000	-4.930161000	-1.682682000
6	0.000000000	2.657370000	-3.035299000
6	1.944794000	2.406760000	-1.631917000
6	2.420659000	3.644160000	-2.066382000
6	1.683930000	4.390268000	-2.986077000
6	0.472904000	3.896725000	-3.469370000
1	-0.107060000	4.479170000	-4.187165000
1	-0.953187000	2.285312000	-3.421745000
1	2.534894000	1.840725000	-0.903475000
1	3.367291000	4.028003000	-1.682682000
1	2.053848000	5.359087000	-3.325051000
6	-3.056712000	0.480861000	-1.631917000
6	-2.301350000	-1.328685000	-3.035299000
6	-3.611115000	-1.538816000	-3.469370000
6	-4.366265000	0.274272000	-2.066382000
6	-4.644049000	-0.736808000	-2.986077000
1	-5.668030000	-0.900859000	-3.325051000
1	-3.825545000	-2.332302000	-4.187165000
1	-2.861562000	1.274920000	-0.903475000
1	-5.171998000	0.902158000	-1.682682000
1	-1.502545000	-1.968140000	-3.421745000

E = -1396.16846299 au

ZPVE = 0.544320 au

Hexaphenyldiplumbane – B3LYP/def2-TZVP

82	0.000000000	0.000000000	1.475092000
82	0.000000000	0.000000000	-1.475092000
6	-1.331951000	-1.619031000	2.315661000
6	-2.269524000	-1.310310000	3.300671000
6	-3.093402000	-2.302703000	3.827000000
6	-2.987569000	-3.611800000	3.371314000
6	-2.056409000	-3.927154000	2.387859000
6	-1.233436000	-2.934649000	1.861536000
1	-2.367648000	-0.294521000	3.666174000
1	-3.817561000	-2.050731000	4.592553000
1	-3.628528000	-4.383144000	3.779992000
6	2.068098000	-0.343987000	2.315661000
6	2.269524000	-1.310310000	3.300671000
6	3.158199000	0.399138000	1.861536000
6	4.429220000	0.182674000	2.387859000
6	3.540900000	-1.527613000	3.827000000
6	4.621695000	-0.781410000	3.371314000
1	5.610178000	-0.950825000	3.779992000
1	5.267544000	0.766986000	2.027791000
1	1.438887000	-1.903183000	3.666174000
1	3.684766000	-2.280739000	4.592553000
1	3.031208000	1.154079000	1.093781000
6	-0.736147000	1.963019000	2.315661000
6	-1.924763000	2.535512000	1.861536000
6	0.000000000	2.620621000	3.300671000
6	-0.447498000	3.830316000	3.827000000
6	-2.372811000	3.744479000	2.387859000
6	-1.634127000	4.393211000	3.371314000
1	-2.515065000	2.048064000	1.093781000
1	-3.298002000	4.178334000	2.027791000
1	0.928761000	2.197704000	3.666174000
1	-1.981650000	5.333969000	3.779992000
1	0.132795000	4.331470000	4.592553000
6	0.736147000	-1.963019000	-2.315661000
6	1.924763000	-2.535512000	-1.861536000
6	2.372811000	-3.744479000	-2.387859000
6	1.634127000	-4.393211000	-3.371314000
6	0.447498000	-3.830316000	-3.827000000
6	0.000000000	-2.620621000	-3.300671000
1	-0.928761000	-2.197704000	-3.666174000
1	1.981650000	-5.333969000	-3.779992000
1	-0.132795000	-4.331470000	-4.592553000
1	2.515065000	-2.048064000	-1.093781000
1	3.298002000	-4.178334000	-2.027791000
6	-2.068098000	0.343987000	-2.315661000
6	-3.158199000	-0.399138000	-1.861536000
6	-4.429220000	-0.182674000	-2.387859000
6	-4.621695000	0.781410000	-3.371314000
6	-3.540900000	1.527613000	-3.827000000
6	-2.269524000	1.310310000	-3.300671000
1	-3.031208000	-1.154079000	-1.093781000
1	-5.267544000	-0.766986000	-2.027791000
1	-5.610178000	0.950825000	-3.779992000
1	-3.684766000	2.280739000	-4.592553000
1	-1.438887000	1.903183000	-3.666174000
6	1.331951000	1.619031000	-2.315661000
6	1.233436000	2.934649000	-1.861536000
6	2.269524000	1.310310000	-3.300671000
6	3.093402000	2.302703000	-3.827000000
6	2.056409000	3.927154000	-2.387859000
6	2.987569000	3.611800000	-3.371314000
1	1.969543000	4.945320000	-2.027791000
1	3.628528000	4.383144000	-3.779992000
1	0.516142000	3.202142000	-1.093781000
1	2.367648000	0.294521000	-3.666174000
1	3.817561000	2.050731000	-4.592553000
1	-0.516142000	-3.202142000	1.093781000
1	-1.969543000	-4.945320000	2.027791000

E = -1776.20440190 au

ZPVE = 0.537351 au

Hexaphenyldiplumbane – B3LYP-D3(BJ)/def2-TZVP

82	0.000000000	0.000000000	1.435482000
82	0.000000000	0.000000000	-1.435482000
6	-1.360166000	-1.598671000	2.200535000
6	-2.291686000	-1.323105000	3.198545000
6	-3.159411000	-2.317372000	3.641933000
6	-3.101736000	-3.590643000	3.087064000
6	-2.176177000	-3.869730000	2.087466000
6	-1.308970000	-2.875995000	1.644223000
1	-2.350903000	-0.331761000	3.632640000
1	-3.881185000	-2.096135000	4.418571000
1	-3.777798000	-4.363097000	3.430876000

6	2.064572000	-0.378603000	2.200535000
6	2.291686000	-1.323105000	3.198545000
6	3.145169000	0.304396000	1.644223000
6	4.439373000	0.050240000	2.087466000
6	3.586609000	-1.577444000	3.641933000
6	4.660456000	-0.890861000	3.087064000
1	5.667452000	-1.090120000	3.430876000
1	5.273576000	0.585082000	1.650492000
1	1.462765000	-1.870062000	3.632640000
1	3.755899000	-2.313137000	4.418571000
1	2.989098000	1.035511000	0.858934000
6	-0.704406000	1.977273000	2.200535000
6	-1.836199000	2.571599000	1.644223000
6	0.000000000	2.646211000	3.198545000
6	-0.427198000	3.894816000	3.641933000
6	-2.263195000	3.819490000	2.087466000
6	-1.558720000	4.481504000	3.087064000
1	-2.391328000	2.070879000	0.858934000
1	-3.143484000	4.274510000	1.650492000
1	0.888138000	2.201823000	3.632640000
1	-1.889654000	5.453218000	3.430876000
1	0.125286000	4.409272000	4.418571000
6	0.704406000	-1.977273000	-2.200535000
6	1.836199000	-2.571599000	-1.644223000
6	2.263195000	-3.819490000	-2.087466000
6	1.558720000	-4.481504000	-3.087064000
6	0.427198000	-3.894816000	-3.641933000
6	0.000000000	-2.646211000	-3.198545000
1	-0.888138000	-2.201823000	-3.632640000
1	1.889654000	-5.453218000	-3.430876000
1	-0.125286000	-4.409272000	-4.418571000
1	2.391328000	-2.070879000	-0.858934000
1	3.143484000	-4.274510000	-1.650492000
6	-2.064572000	0.378603000	-2.200535000
6	-3.145169000	-0.304396000	-1.644223000
6	-4.439373000	-0.050240000	-2.087466000
6	-4.660456000	0.890861000	-3.087064000
6	-3.586609000	1.577444000	-3.641933000
6	-2.291686000	1.323105000	-3.198545000
1	-2.989098000	-1.035511000	-0.858934000
1	-5.273576000	-0.585082000	-1.650492000
1	-5.667452000	1.090120000	-3.430876000
1	-3.755899000	2.313137000	-4.418571000
1	-1.462765000	1.870062000	-3.632640000
6	1.360166000	1.598671000	-2.200535000
6	1.308970000	2.875995000	-1.644223000
6	2.291686000	1.323105000	-3.198545000
6	3.159411000	2.317372000	-3.641933000
6	2.176177000	3.869730000	-2.087466000
6	3.101736000	3.590643000	-3.087064000
1	2.130092000	4.859592000	-1.650492000
1	3.777798000	4.363097000	-3.430876000
1	0.597770000	3.106390000	-0.858934000
1	2.350903000	0.331761000	-3.632640000
1	3.881185000	2.096135000	-4.418571000
1	-0.597770000	-3.106390000	0.858934000
1	-2.130092000	-4.859592000	1.650492000

E = -1776.38829462 au

ZPVE = 0.538097 au

Hexaphenyldiplumbane – M06-2X/cc-pVDZ/SDD

82	-1.445891000	-0.012481000	0.013859000
82	1.444120000	-0.029667000	0.001883000
6	-2.250691000	-0.774221000	-1.916758000
6	-1.793734000	-0.251603000	-3.134497000
6	-2.287813000	-0.736878000	-4.346364000
6	-3.241610000	-1.754027000	-4.354048000
6	-3.700123000	-2.284046000	-3.148889000
6	-3.206985000	-1.796865000	-1.936981000
1	-1.042788000	0.544230000	-3.150468000
1	-1.924518000	-0.319186000	-5.286770000
1	-3.626876000	-2.135010000	-5.300945000
1	-4.445505000	-3.081089000	-3.150293000
1	-3.575686000	-2.223629000	-0.999935000
6	-2.166997000	2.075791000	0.293343000
6	-2.273506000	-1.265510000	1.656622000
6	-1.867238000	-2.597793000	1.812579000
6	-3.197944000	-0.730898000	2.562432000
6	-2.380117000	-3.381124000	2.847709000
6	-3.302179000	-2.838658000	3.742150000
6	-3.709764000	-1.513148000	3.599382000
1	-3.526374000	0.307609000	2.464338000
1	-1.141292000	-3.040845000	1.124026000
1	-2.056343000	-4.417591000	2.955806000
1	-3.702512000	-3.449722000	4.552385000
1	-4.430095000	-1.084152000	4.297944000
6	-1.681468000	2.858832000	1.349896000
6	-3.089294000	2.640230000	-0.596412000
6	-3.522198000	3.957219000	-0.430379000
6	-3.036411000	4.724961000	0.626986000
6	-2.115268000	4.174899000	1.517755000
1	-0.954443000	2.448230000	2.057406000
1	-3.477795000	2.051570000	-1.432171000
1	-4.241710000	4.384279000	-1.131005000
1	-3.374433000	5.754158000	0.756155000
1	-1.730163000	4.771794000	2.346088000
6	2.183264000	1.322421000	-1.604859000
6	2.256661000	-2.067961000	-0.368031000
6	2.246323000	0.704222000	1.944603000
6	3.162627000	1.762683000	1.980248000
6	3.654769000	2.235115000	3.198378000
6	1.827462000	0.131622000	3.153357000
6	2.320594000	0.602099000	4.371418000
6	3.235141000	1.654562000	4.394370000
1	3.499749000	2.229570000	1.050503000
1	3.619574000	2.024008000	5.346165000
1	4.368840000	3.060262000	3.211768000
1	1.106029000	-0.691082000	3.157319000
1	1.986814000	0.145702000	5.304729000
6	3.151803000	0.886932000	-2.517596000
6	1.670345000	2.620894000	-1.731036000
6	2.122550000	3.468123000	-2.744026000
6	3.089679000	3.024445000	-3.645321000
6	3.603062000	1.733275000	-3.532175000
1	4.358598000	1.381309000	-4.236584000
1	3.561772000	-0.124183000	-2.443404000
1	0.906785000	2.986846000	-1.037464000
1	1.715424000	4.476900000	-2.829591000
1	3.442250000	3.685713000	-4.438211000
6	1.798721000	-2.830636000	-1.451008000
6	3.223344000	-2.616102000	0.483743000
6	3.726566000	-3.898125000	0.254522000
6	2.302762000	-4.111600000	-1.681753000
6	3.267519000	-4.646066000	-0.828637000
1	3.660703000	-5.647908000	-1.007325000
1	4.480481000	-4.312998000	0.925593000
1	1.038863000	-2.431448000	-2.129667000
1	1.938295000	-4.693501000	-2.529849000
1	3.592461000	-2.042082000	1.338280000

E = -1396.19973386 au

ZPVE = 0.542796 au

Triphenyl(triphenylmethyl)silane – B3LYP/def2-TZVP

6	0.000000000	0.000000000	-1.200003000
14	0.000000000	0.000000000	0.811239000
6	-0.832746000	1.206513000	-1.697200000
6	-1.857626000	1.072501000	-2.638261000

6	-2.578208000	2.173336000	-3.093946000
6	-2.291382000	3.447638000	-2.627244000
6	-1.260480000	3.606917000	-1.707947000
6	-0.543712000	2.506542000	-1.257604000
1	-2.106425000	0.099157000	-3.032434000
1	-3.366181000	2.024736000	-3.822125000
1	-2.853550000	4.303620000	-2.978655000
6	1.461244000	0.117922000	-1.697200000
6	1.857626000	1.072501000	-2.638261000
6	2.442585000	-0.782403000	-1.257604000
6	3.753921000	-0.711851000	-1.707947000
6	3.171268000	1.146126000	-3.093946000
6	4.131433000	0.260576000	-2.627244000
1	5.153819000	0.319437000	-2.978655000
1	4.480933000	-1.424159000	-1.337598000
1	1.139085000	1.774639000	-3.032434000
1	3.436563000	1.902830000	-3.822125000
1	2.181760000	-1.562160000	-0.558196000
6	-0.628498000	-1.324435000	-1.697200000
6	-1.898873000	-1.724139000	-1.257604000
6	0.000000000	-2.145002000	-2.638261000
6	-0.593060000	-3.319461000	-3.093946000
6	-2.493442000	-2.895066000	-1.707947000
6	-1.840051000	-3.708215000	-2.627244000
1	-2.443751000	-1.108380000	-0.558196000
1	-3.473824000	-3.168522000	-1.337598000
1	0.967340000	-1.873796000	-3.032434000
1	-2.300269000	-4.623057000	-2.978655000
1	-0.070382000	-3.927566000	-3.822125000
6	0.949587000	1.486933000	1.528561000
6	2.274925000	1.793355000	1.179020000
6	2.953155000	2.856854000	1.762760000
6	2.327810000	3.645641000	2.722046000
6	1.021070000	3.357515000	3.093098000
6	0.345326000	2.293266000	2.503975000
1	-0.671746000	2.092791000	2.813059000
1	2.855951000	4.474494000	3.177184000
1	0.522666000	3.959979000	3.842831000
1	2.792733000	1.198713000	0.439518000
1	3.973754000	3.067370000	1.467091000
6	-1.762515000	0.078900000	1.528561000
6	-2.690554000	1.073465000	1.179020000
6	-3.950686000	1.129080000	1.762760000
6	-4.321123000	0.193123000	2.722046000
6	-3.418228000	-0.794485000	3.093098000
6	-2.158690000	-0.847572000	2.503975000
1	-2.434483000	1.819221000	0.439518000
1	-4.643297000	1.907687000	1.467091000
1	-5.303002000	0.236079000	3.177184000
1	-3.690775000	-1.527347000	3.842831000
1	-1.476537000	-1.628144000	2.813059000
6	0.812929000	-1.565833000	1.528561000
6	0.415629000	-2.866820000	1.179020000
6	1.813364000	-1.445694000	2.503975000
6	2.397158000	-2.563030000	3.093098000
6	0.997531000	-3.985934000	1.762760000
6	1.993312000	-3.838763000	2.722046000
1	0.669543000	-4.975057000	1.467091000
1	2.447050000	-4.710574000	3.177184000
1	-0.358250000	-3.017934000	0.439518000
1	2.148283000	-0.464647000	2.813059000
1	3.168109000	-2.432632000	3.842831000
1	0.261990000	2.670540000	-0.558196000
1	-1.007109000	4.592681000	-1.337598000

E = -1718.04490112 au

ZPVE = 0.553902 au

Triphenyl(triphenylmethyl)silane – B3LYP-D3(BJ)/def2-TZVP

6	0.000000000	0.000000000	-1.189503000
14	0.000000000	0.000000000	0.789482000
6	-0.819108000	1.213897000	-1.654603000
6	-1.899505000	1.096680000	-2.528885000
6	-2.644836000	2.206978000	-2.913338000
6	-2.322506000	3.470529000	-2.441319000
6	-1.227336000	3.612020000	-1.596349000
6	-0.487348000	2.502831000	-1.216424000
1	-2.175368000	0.129009000	-2.917508000
1	-3.481468000	2.075203000	-3.587782000
1	-2.906307000	4.333426000	-2.734353000
6	1.460819000	0.102420000	-1.654603000
6	1.899505000	1.096680000	-2.528885000
6	2.411189000	-0.829360000	-1.216424000
6	3.741769000	-0.743106000	-1.596349000
6	3.233717000	1.187006000	-2.913338000
6	4.166819000	0.276084000	-2.441319000
1	5.206011000	0.350223000	-2.734353000
1	4.447436000	-1.476132000	-1.226650000
1	1.199409000	1.819419000	-2.917508000
1	3.537912000	1.977439000	-3.587782000
1	2.110650000	-1.636981000	-0.568520000
6	-0.641711000	-1.316317000	-1.654603000
6	-1.923841000	-1.673471000	-1.216424000
6	0.000000000	-2.193360000	-2.528885000
6	-0.588881000	-3.393984000	-2.913338000
6	-2.514434000	-2.868914000	-1.596349000
6	-1.844314000	-3.746614000	-2.441319000
1	-2.472992000	-1.009386000	-0.568520000
1	-3.502086000	-3.113527000	-1.226650000
1	0.975959000	-1.948429000	-2.917508000
1	-2.299704000	-4.683649000	-2.734353000
1	-0.056444000	-4.052641000	-3.587782000
6	0.968821000	1.466188000	1.473094000
6	2.300799000	1.729327000	1.121134000
6	2.984914000	2.814819000	1.651537000
6	2.357317000	3.664339000	2.555939000
6	1.043632000	3.414162000	2.929843000
6	0.361333000	2.327525000	2.394371000
1	-0.662474000	2.153272000	2.695864000
1	2.890220000	4.511949000	2.968013000
1	0.546524000	4.064887000	3.638346000
1	2.813800000	1.087079000	0.420675000
1	4.010728000	2.996878000	1.356665000
6	-1.754167000	0.105929000	1.473094000
6	-2.648041000	1.127887000	1.121134000
6	-3.930162000	1.177602000	1.651537000
6	-4.352069000	0.209327000	2.555939000
6	-3.478567000	-0.803269000	2.929843000
6	-2.196362000	-0.850839000	2.394371000
1	-2.348338000	1.893283000	0.420675000
1	-4.600736000	1.974953000	1.356665000
1	-5.352573000	0.247029000	2.968013000
1	-3.793557000	-1.559140000	3.638346000
1	-1.533551000	-1.650356000	2.695864000
6	0.785346000	-1.572117000	1.473094000
6	0.347242000	-2.857214000	1.121134000
6	1.835029000	-1.476686000	2.394371000
6	2.434935000	-2.610893000	2.929843000
6	0.945248000	-3.992421000	1.651537000
6	1.994752000	-3.873666000	2.555939000
1	0.590008000	-4.971831000	1.356665000
1	2.462353000	-4.758979000	2.968013000
1	-0.465462000	-2.980362000	0.420675000
1	2.196025000	-0.502916000	2.695864000
1	3.247034000	-2.505747000	3.638346000
1	0.362342000	2.646367000	-0.568520000
1	-0.945350000	4.589659000	-1.226650000

E = -1718.25232778 au

ZPVE = 0.555671 au

Triphenyl(triphenylmethyl)silane – M06-2X/cc-pVDZ/SDD

6	0.000000000	0.000000000	-1.206573000
14	0.000000000	0.000000000	0.780291000
6	1.427242000	-0.347915000	-1.664515000
6	-0.412318000	1.409985000	-1.664515000
6	-1.014924000	-1.062070000	-1.664515000
6	1.706972000	-1.423585000	-2.515848000
6	2.518282000	0.430858000	-1.233632000
6	3.825163000	0.116265000	-1.593279000
6	4.086903000	-0.986023000	-2.407933000
6	3.018275000	-1.742602000	-2.876609000
1	0.895904000	-2.032440000	-2.910932000
1	2.345026000	1.308346000	-0.611757000
1	4.643210000	0.740396000	-1.230852000
1	5.110677000	-1.240376000	-2.684718000
1	3.196441000	-2.593816000	-3.535270000
6	0.379375000	2.190074000	-2.515848000
6	-1.632275000	1.965468000	-1.233632000
6	-2.013270000	3.254556000	-1.593279000
6	-1.189531000	4.032374000	-2.407933000
6	0.000000000	3.485204000	-2.876609000
1	1.312193000	1.792095000	-2.910932000
1	-2.305574000	1.376679000	-0.611757000
1	-2.962807000	3.650939000	-1.230852000
1	-1.481141000	5.046165000	-2.684718000
1	0.648090000	4.065107000	-3.535270000
6	-2.086347000	-0.766489000	-2.515848000
6	-0.886008000	-2.396325000	-1.233632000
6	-3.018275000	-1.742602000	-2.876609000
6	-2.897372000	-3.046351000	-2.407933000
6	-1.811893000	-3.370821000	-1.593279000
1	-2.208096000	0.240345000	-2.910932000
1	-3.844531000	-1.471291000	-3.535270000
1	-3.629536000	-3.805788000	-2.684718000
1	-1.680403000	-4.391336000	-1.230852000
1	-0.039452000	-2.685025000	-0.611757000
6	1.035467000	1.436854000	1.469970000
6	-1.762085000	0.178313000	1.469970000
6	0.726619000	-1.615167000	1.469970000
6	2.066679000	1.167093000	2.384227000
6	2.852847000	2.191112000	2.915061000
6	2.622360000	3.512906000	2.543023000
6	0.809475000	2.781956000	1.118484000
6	1.594629000	3.805480000	1.645912000
1	3.648772000	1.951182000	3.621732000
1	3.237901000	4.314994000	2.953201000
1	2.266202000	0.137728000	2.688062000
1	0.012026000	3.043100000	0.420498000
1	1.400624000	4.838315000	1.352652000
6	-0.022607000	-2.373343000	2.384227000
6	2.004508000	-2.092004000	1.118484000
6	2.498328000	-3.283730000	1.645912000
6	0.471135000	-3.566194000	2.915061000
1	-1.013825000	-2.031453000	2.688062000
1	2.629389000	-1.531965000	0.420498000
6	1.731085000	-4.027484000	2.543023000
1	3.489792000	-3.632134000	1.352652000
1	-0.134612000	-4.135521000	3.621732000
1	2.117944000	-4.961602000	2.953201000
6	-2.813982000	-0.689952000	1.118484000
6	-2.044072000	1.206250000	2.384227000
6	-3.323982000	1.375082000	2.915061000
6	-4.092957000	-0.521751000	1.645912000
6	-4.353446000	0.514578000	2.543023000
1	-1.252377000	1.893725000	2.688062000
1	-2.641415000	-1.511135000	0.420498000
1	-4.890416000	-1.206181000	1.352652000
1	-5.355845000	0.646608000	2.953201000
1	-3.514160000	2.184338000	3.621732000

E = -1717.05904177 au

ZPVE = 0.558972 au

6.2. Ph_3T Radical Structures

Triphenylmethyl radical – B3LYP/def2-TZVP

6	0.000000000	0.000000000	-0.000562000
6	0.000000000	1.459958000	-0.000402000
6	-0.989426000	2.195290000	-0.684709000
6	-0.984797000	3.580942000	-0.687438000
6	-0.000110000	4.284800000	0.000412000
6	0.984652000	3.580629000	0.687853000
6	0.989357000	2.194985000	0.684325000
1	-1.753189000	1.664510000	-1.236865000
1	-1.750029000	4.116241000	-1.236062000
1	-0.000174000	5.367330000	0.000733000
6	1.264361000	-0.729979000	-0.000402000
6	2.395890000	-0.240777000	-0.684709000
6	1.406234000	-1.954301000	0.684325000
6	2.608589000	-2.643048000	0.687853000
6	3.593586000	-0.937611000	-0.687438000
6	3.710801000	-2.142305000	0.000412000
1	4.648331000	-2.683514000	0.000733000
1	2.689376000	-3.573223000	1.236815000
1	2.318102000	0.686051000	-1.236865000
1	4.439784000	-0.542551000	-1.236062000
1	0.564471000	-2.350203000	1.236259000
6	-1.264361000	-0.729979000	-0.000402000
6	-2.395591000	-0.240684000	0.684325000
6	-1.406464000	-1.954513000	-0.684709000
6	-2.608788000	-2.643331000	-0.687438000
6	-3.593242000	-0.937581000	0.687853000
6	-3.710691000	-2.142495000	0.000412000
1	-2.317571000	0.686255000	1.236259000
1	-4.439189000	-0.542456000	1.236815000
1	-0.564913000	-2.350561000	-1.236865000
1	-4.648157000	-2.683816000	0.000733000
1	-2.689755000	-3.573690000	-1.236062000
1	1.753100000	1.663948000	1.236259000
1	1.749814000	4.115679000	1.236815000

E = -733.296692867 au

ZPVE = 0.277228 au

Triphenylmethyl radical – B3LYP-D3(BJ)/def2-TZVP

6	0.000000000	0.000000000	-0.000770000
6	0.000000000	1.454837000	-0.000544000
6	-0.998465000	2.186945000	-0.672004000
6	-0.994293000	3.571547000	-0.673943000
6	-0.000071000	4.274492000	0.000547000
6	0.994201000	3.571090000	0.674501000
6	0.998402000	2.186498000	0.671497000
1	-1.767552000	1.652875000	-1.212447000
1	-1.766355000	4.107370000	-1.211539000
1	-0.000129000	5.356617000	0.000975000
6	1.259925000	-0.727418000	-0.000544000
6	2.393183000	-0.228776000	-0.672004000
6	1.394362000	-1.957891000	0.671497000
6	2.595555000	-2.646548000	0.674501000
6	3.590197000	-0.924690000	-0.673943000
6	3.701854000	-2.137185000	0.000547000
1	4.639031000	-2.678197000	0.000975000
1	2.673277000	-3.582839000	1.212564000
1	2.315208000	0.704308000	-1.212447000
1	4.440264000	-0.523977000	-1.211539000
1	0.547007000	-2.356644000	1.211667000
6	-1.259925000	-0.727418000	-0.000544000
6	-2.392764000	-0.228607000	0.671497000
6	-1.394717000	-1.958169000	-0.672004000
6	-2.595904000	-2.646857000	-0.673943000
6	-3.589755000	-0.924542000	0.674501000
6	-3.701783000	-2.137308000	0.000547000
1	-2.314417000	0.704600000	1.211667000
1	-4.439468000	-0.523707000	1.212564000
1	-0.547656000	-2.357183000	-1.212447000
1	-4.638902000	-2.678420000	0.000975000
1	-2.673909000	-3.583393000	-1.211539000
1	1.767410000	1.652044000	1.211667000
1	1.766191000	4.106546000	1.212564000

E = -733.377529494 au

ZPVE = 0.277761 au

Triphenylmethyl radical – M06-2X/cc-pVDZ/SDD

6	0.000000000	0.000000000	0.001701000
6	0.000000000	1.462386000	0.001154000
6	-1.266463000	-0.731193000	0.001154000
6	1.266463000	-0.731193000	0.001154000
6	1.005588000	2.193613000	0.669225000
6	-2.402519000	-0.225942000	0.669225000
6	-1.396286000	-1.967100000	-0.668065000
6	-2.602210000	-2.658370000	-0.671001000
6	-3.713358000	-2.143425000	-0.001243000
6	-3.604293000	-0.924300000	0.669779000
1	-0.538253000	-2.369924000	-1.208043000
1	-2.678372000	-3.604999000	-1.207765000
1	-4.657867000	-2.688461000	-0.002206000
1	-4.462865000	-0.517098000	1.205568000
1	-2.322888000	0.718177000	1.209952000
6	1.396931000	-1.967671000	0.669225000
6	2.401702000	-0.225669000	-0.668065000
6	3.603321000	-0.924395000	-0.671001000
6	2.602614000	-2.659260000	0.669779000
6	3.712939000	-2.144150000	-0.001243000
1	2.321541000	0.718822000	-1.208043000
1	4.657209000	-2.689601000	-0.002206000
1	4.461207000	-0.517038000	-1.207765000
1	0.539485000	-2.370768000	1.209952000
1	2.679252000	-3.606405000	1.205568000
6	-1.005416000	2.192770000	-0.668065000
6	-1.001111000	3.582765000	-0.671001000
6	0.000419000	4.287574000	-0.001243000
6	1.001679000	3.583559000	0.669779000
1	-1.783288000	1.651103000	-1.208043000
1	-1.782835000	4.122038000	-1.207765000
1	0.000658000	5.378061000	-0.002206000
1	1.783403000	1.652591000	1.209952000
1	1.783613000	4.123503000	1.205568000

E = -732.793730789 au

ZPVE = 0.280076 au

Triphenylsilyl radical – B3LYP/def2-TZVP

14	0.000000000	0.000000000	0.737251000
6	0.000000000	1.808928000	0.252259000
6	-1.011429000	2.662915000	0.723605000
6	-1.023822000	4.013273000	0.401963000
6	-0.014889000	4.550523000	-0.391949000
6	1.001252000	3.726504000	-0.862343000
6	1.008615000	2.373407000	-0.543853000
1	-1.798655000	2.266712000	1.355198000
1	-1.816790000	4.649338000	0.775988000
1	-0.020356000	5.604644000	-0.639843000
1	1.788585000	4.136842000	-1.482984000
1	1.805111000	1.747681000	-0.926958000
6	-1.566577000	-0.904464000	0.252259000
6	1.566577000	-0.904464000	0.252259000
6	2.811867000	-0.455534000	0.723605000
6	1.551123000	-2.060190000	-0.543853000
6	3.987508000	-1.119981000	0.401963000
6	3.948313000	-2.262367000	-0.391949000
6	2.726621000	-2.730361000	-0.862343000
1	0.610981000	-2.437112000	-0.926958000
1	2.862358000	0.424325000	1.355198000
1	4.934840000	-0.751282000	0.775988000
1	4.863943000	-2.784693000	-0.639843000
1	2.688318000	-3.617381000	-1.482984000
6	-1.800438000	-2.207381000	0.723605000
6	-2.559738000	-0.313217000	-0.543853000
6	-3.727873000	-0.996142000	-0.862343000
6	-3.933424000	-2.288156000	-0.391949000
6	-2.963686000	-2.893293000	0.401963000
1	-1.063703000	-2.691037000	1.355198000
1	-2.416092000	0.689431000	-0.926958000
1	-4.476903000	-0.519461000	-1.482984000
1	-4.843586000	-2.819951000	-0.639843000
1	-3.118049000	-3.898055000	0.775988000

E = -984.696555539 au

ZPVE = 0.271852 au

Triphenylsilyl radical – B3LYP-D3(BJ)/def2-TZVP

14	0.000000000	0.000000000	0.810347000
6	0.000000000	1.796479000	0.292210000
6	-1.033386000	2.651682000	0.708105000
6	-1.052174000	3.989228000	0.340634000
6	-0.028190000	4.510821000	-0.443685000
6	1.009331000	3.684492000	-0.859051000
6	1.023412000	2.343890000	-0.495484000
1	-1.833028000	2.263863000	1.328532000
1	-1.862014000	4.627896000	0.670245000
1	-0.039057000	5.555359000	-0.727603000
1	1.807440000	4.083497000	-1.472573000
1	1.833360000	1.712079000	-0.836595000
6	-1.555797000	-0.898240000	0.292210000
6	1.555797000	-0.898240000	0.292210000
6	2.813117000	-0.430902000	0.708105000
6	1.518162000	-2.058246000	-0.495484000
6	3.980860000	-1.083405000	0.340634000
6	3.920581000	-2.230997000	-0.443685000
6	2.686198000	-2.716352000	-0.859051000
1	0.566024000	-2.443776000	-0.836595000

1	2.877077000	0.455517000	1.328532000
1	4.938883000	-0.701397000	0.670245000
1	4.830610000	-2.743855000	-0.727603000
1	2.632693000	-3.607037000	-1.472573000
6	-1.779731000	-2.220780000	0.708105000
6	-2.541574000	-0.285644000	-0.495484000
6	-3.695529000	-0.968140000	-0.859051000
6	-3.892390000	-2.279824000	-0.443685000
6	-2.928686000	-2.905823000	0.340634000
1	-1.044050000	-2.719380000	1.328532000
1	-2.399384000	0.731696000	-0.836595000
1	-4.440132000	-0.476460000	-1.472573000
1	-4.791553000	-2.811504000	-0.727603000
1	-3.076869000	-3.926499000	0.670245000

E = -984.779672636 au

ZPVE = 0.272320 au

Triphenylsilyl radical – M06-2X/cc-pVDZ/SDD

14	0.000000000	0.000000000	0.818202000
6	0.000000000	1.802754000	0.289181000
6	-1.561231000	-0.901377000	0.289181000
6	1.561231000	-0.901377000	0.289181000
6	1.033157000	2.656084000	0.717497000
6	-2.816815000	-0.433302000	0.717497000
6	-1.523220000	-2.057176000	-0.508438000
6	-2.697439000	-2.716513000	-0.870079000
6	-3.932977000	-2.232372000	-0.442572000
6	-3.991208000	-1.086186000	0.351352000
1	-0.562612000	-2.441983000	-0.858619000
1	-2.647180000	-3.610490000	-1.493570000
1	-4.851320000	-2.748202000	-0.726475000
1	-4.954993000	-0.703970000	0.690909000
1	-2.875498000	0.458649000	1.347842000
6	1.783658000	-2.222783000	0.717497000
6	2.543177000	-0.290559000	-0.508438000
6	3.701289000	-0.977794000	-0.870079000
6	2.936269000	-2.913395000	0.351352000
6	3.899780000	-2.289872000	-0.442572000
1	2.396125000	0.733755000	-0.858619000
1	4.805673000	-2.827266000	-0.726475000
1	4.450366000	-0.487281000	-1.493570000
1	1.040547000	-2.719578000	1.347842000
1	3.087152000	-3.939165000	0.690909000
6	-1.019957000	2.347735000	-0.508438000
6	-1.003850000	3.694307000	-0.870079000
6	0.033197000	4.522244000	-0.442572000
6	1.054939000	3.999581000	0.351352000
1	-1.833513000	1.708228000	-0.858619000
1	-1.803186000	4.097770000	-1.493570000
1	0.045647000	5.575467000	-0.726475000
1	1.834950000	2.260930000	1.347842000
1	1.867841000	4.643135000	0.690909000

E = -984.167133268 au

ZPVE = 0.274416 au

Triphenylgermanyl radical – B3LYP/def2-TZVP

32	0.000000000	0.000000000	0.760303000
6	0.000000000	1.882234000	0.189431000
6	-1.630062000	-0.941117000	0.189431000
6	1.630062000	-0.941117000	0.189431000
6	2.591271000	-0.323239000	-0.618945000
6	3.750135000	-0.997134000	-0.989721000
6	1.873526000	-2.248956000	0.627865000
6	3.029427000	-2.924360000	0.255391000
6	3.971941000	-2.298947000	-0.554632000
1	2.433118000	0.688261000	-0.972353000
1	4.874966000	-2.822657000	-0.842570000
1	4.479240000	-0.504753000	-1.621818000
1	1.155734000	-2.747898000	1.269150000
1	3.197370000	-3.937029000	0.601358000
6	-1.015702000	2.405726000	-0.618945000
6	1.010890000	2.746999000	0.627865000
6	1.017856000	4.085741000	0.255391000
6	0.004976000	4.589275000	-0.554632000
6	-1.011524000	3.746279000	-0.989721000
1	-1.802491000	4.131512000	-1.621818000
1	-1.812611000	1.763011000	-0.972353000
1	1.801882000	2.374844000	1.269150000
1	1.810882000	4.737518000	0.601358000
1	0.007010000	5.633173000	-0.842570000
6	-2.884416000	-0.498043000	0.627865000
6	-1.575569000	-2.082486000	-0.618945000
6	-2.738611000	-2.749145000	-0.989721000
6	-4.047283000	-1.161381000	0.255391000
6	-3.976916000	-2.290328000	-0.554632000
1	-4.881976000	-2.810516000	-0.842570000
1	-2.676749000	-3.626759000	-1.621818000
1	-2.957617000	0.373054000	1.269150000
1	-5.008252000	-0.800490000	0.601358000
1	-0.620507000	-2.451272000	-0.972353000

E = -2772.21989764 au

ZPVE = 0.270433 au

Triphenylgermanyl radical – B3LYP-D3(BJ)/def2-TZVP

32	0.000000000	0.000000000	0.835022000
6	1.330250000	1.307285000	0.225663000
6	-1.797267000	0.498388000	0.225663000
6	0.467017000	-1.805673000	0.225663000
6	1.589528000	-2.034607000	-0.576842000
6	1.909382000	-3.319382000	-0.999949000
6	-0.316691000	-2.901785000	0.604339000
6	0.000000000	-4.185169000	0.178775000
6	1.115699000	-4.396988000	-0.624328000
1	2.211479000	-1.203903000	-0.885173000
1	1.365725000	-5.397295000	-0.953950000
1	2.777731000	-3.478275000	-1.627193000
1	-1.184129000	-2.753000000	1.236774000
1	-0.620660000	-5.020957000	0.476689000
6	0.967257000	2.393875000	-0.576842000
6	2.671365000	1.176630000	0.604339000
6	3.624462000	2.092584000	0.178775000
6	3.250054000	3.164718000	-0.624328000
6	1.919978000	3.313264000	-0.999949000
1	1.623409000	4.144723000	-1.627193000
1	-0.063129000	2.517148000	-0.885173000
1	2.976232000	0.351014000	1.236774000
1	4.658606000	1.972972000	0.476689000
1	3.991332000	3.881400000	-0.953950000
6	-2.354674000	1.725155000	0.604339000
6	-2.556786000	-0.359268000	-0.576842000
6	-3.829360000	0.006118000	-0.999949000
6	-3.624462000	2.092584000	0.178775000
6	-4.365753000	1.232271000	-0.624328000
1	-5.357057000	1.515895000	-0.953950000
1	-4.401140000	-0.666448000	-1.627193000
1	-1.792103000	2.401986000	1.236774000
1	-4.037947000	3.047985000	0.476689000
1	-2.148350000	-1.313245000	-0.885173000

E = -2772.30160329 au

ZPVE = 0.270892 au

Triphenylgermanyl radical – M06-2X/cc-pVDZ/SDD

32	0.000000000	0.000000000	0.805624000
6	-1.800588000	0.498739000	0.212339000
6	0.468373000	-1.808724000	0.212339000
6	1.332215000	1.309985000	0.212339000
6	-2.354368000	1.727405000	0.607413000
6	-0.318792000	-2.902645000	0.607413000
6	1.594786000	-2.050839000	-0.588332000
6	1.918370000	-3.346948000	-0.989559000
6	1.121789000	-4.421399000	-0.596155000
6	0.000000000	-4.197353000	0.202516000
1	2.222348000	-1.216530000	-0.911055000
1	2.795072000	-3.517621000	-1.616284000
1	1.375436000	-5.434668000	-0.910752000
1	-0.625434000	-5.034928000	0.515017000
1	-1.195961000	-2.741297000	1.240421000
6	2.673160000	1.175240000	0.607413000
6	0.978686000	2.406545000	-0.588332000
6	1.939357000	3.334831000	-0.989559000
6	3.635014000	2.098677000	0.202516000
6	3.268149000	3.182197000	-0.596155000
1	-0.057628000	2.532875000	-0.911055000
1	4.018842000	3.908497000	-0.910752000
1	1.648813000	4.179413000	-1.616284000
1	2.972013000	0.334916000	1.240421000
1	4.673092000	1.975822000	0.515017000
6	-2.573472000	-0.355706000	-0.588332000
6	-3.857727000	0.012116000	-0.989559000
6	-4.389938000	1.239202000	-0.596155000
6	-3.635014000	2.098677000	0.202516000
1	-2.164720000	-1.316345000	-0.911055000
1	-4.443885000	-0.661793000	-1.616284000
1	-5.394279000	1.526171000	-0.910752000
1	-1.776053000	2.406381000	1.240421000
1	-4.047659000	3.059106000	0.515017000

E = -2771.67971505 au

ZPVE = 0.272896 au

Triphenylstannyl radical – B3LYP/def2-TZVP

50	0.000000000	0.000000000	0.888652000
6	0.469328000	1.987980000	0.135548000
6	-0.298417000	3.093909000	0.513991000
6	0.000000000	4.366501000	0.037661000
6	1.080379000	4.554149000	-0.817805000
6	1.858666000	3.465382000	-1.194467000
6	1.557463000	2.192518000	-0.718290000
1	-1.140806000	2.971433000	1.185960000
1	-0.608710000	5.211260000	0.337043000
1	1.315924000	5.544662000	-1.186849000
1	2.701624000	3.605258000	-1.860599000
1	2.175186000	1.356896000	-1.026244000
6	-1.956305000	-0.587540000	0.135548000
6	1.486977000	-1.400440000	0.135548000
6	2.828613000	-1.288518000	0.513991000
6	1.120045000	-2.445061000	-0.718290000
6	3.781501000	-2.183251000	0.037661000
6	3.403820000	-3.212710000	-0.817805000
6	2.071776000	-3.342343000	-1.194467000
1	0.087514000	-2.562214000	-1.026244000
1	3.143739000	-0.497750000	1.185960000
1	4.817438000	-2.078472000	0.337043000
1	4.143856000	-3.911954000	-1.186849000
1	1.771433000	-4.142304000	-1.860599000
6	-2.530196000	-1.805392000	0.513991000
6	-2.677508000	0.252543000	-0.718290000
6	-3.930441000	-0.123039000	-1.194467000
6	-4.484198000	-1.341439000	-0.817805000
6	-3.781501000	-2.183251000	0.037661000
1	-2.002934000	-2.473683000	1.185960000
1	-2.262699000	1.205318000	-1.026244000
1	-4.473057000	0.537046000	-1.860599000
1	-5.459780000	-1.632707000	-1.186849000
1	-4.208728000	-3.132788000	0.337043000

E = -909.532893569 au

ZPVE = 0.268825 au

Triphenylstannyl radical – B3LYP-D3(BJ)/def2-TZVP

50	0.000000000	0.000000000	0.979920000
6	0.486692000	1.956280000	0.173018000
6	-0.298490000	3.074356000	0.467228000
6	0.000000000	4.316969000	-0.080368000
6	1.097153000	4.460768000	-0.922357000
6	1.892085000	3.358701000	-1.215030000
6	1.590802000	2.115425000	-0.668692000
1	-1.155326000	2.982094000	1.124980000
1	-0.621788000	5.172789000	0.151720000
1	1.332333000	5.428352000	-1.347086000
1	2.747020000	3.465488000	-1.871306000
1	2.216199000	1.265161000	-0.913090000
6	-1.937534000	-0.556653000	0.173018000
6	1.450842000	-1.399627000	0.173018000
6	2.811716000	-1.278678000	0.467228000
6	1.036610000	-2.435388000	-0.668692000
6	3.738605000	-2.158485000	-0.080368000
6	3.314562000	-3.180547000	-0.922357000
6	1.962678000	-3.317944000	-1.215030000
1	-0.012438000	-2.551866000	-0.913090000
1	3.160232000	-0.490505000	1.124980000
1	4.790661000	-2.047910000	0.151720000
1	4.034924000	-3.868010000	-1.347086000
1	1.627691000	-4.111734000	-1.871306000
6	-2.513226000	-1.795678000	0.467228000
6	-2.627413000	0.319963000	-0.668692000
6	-3.854763000	-0.040757000	-1.215030000
6	-4.411715000	-1.280221000	-0.922357000
6	-3.738605000	-2.158485000	-0.080368000
1	-2.004906000	-2.491589000	1.124980000
1	-2.203762000	1.286704000	-0.913090000
1	-4.374711000	0.646245000	-1.871306000
1	-5.367257000	-1.560342000	-1.347086000
1	-4.168873000	-3.124879000	0.151720000

E = -909.614539523 au

ZPVE = 0.269237 au

Triphenylstannyl radical – M06-2X/cc-pVDZ/SDD

50	0.000000000	0.000000000	0.925745000
6	1.942170000	-0.557499000	0.154170000
6	-0.488277000	1.960718000	0.154170000
6	-1.453893000	-1.403220000	0.154170000
6	2.508594000	-1.805722000	0.455621000
6	0.309504000	3.075367000	0.455621000
6	-1.617644000	2.148222000	-0.657008000
6	-1.932045000	3.410079000	-1.162459000
6	-1.123431000	4.505393000	-0.861628000
6	0.000000000	4.336787000	-0.052720000
1	-2.258232000	1.298959000	-0.910888000
1	-2.810939000	3.537558000	-1.796575000
1	-1.369141000	5.491958000	-1.257188000
1	0.635141000	5.191239000	0.186625000
1	1.191809000	2.965380000	1.093092000
6	-2.818098000	-1.269645000	0.455621000
6	-1.051592000	-2.475031000	-0.657008000

6	-1.987193000	-3.378240000	-1.162459000
6	-3.755767000	-2.168393000	-0.052720000
6	-3.340069000	-3.225616000	-0.861628000
1	0.004184000	-2.605166000	-0.910888000
1	-4.071605000	-3.931689000	-1.257188000
1	-1.658145000	-4.203124000	-1.796575000
1	-3.163999000	-0.450553000	1.093092000
1	-4.813315000	-2.045571000	0.186625000
6	2.669236000	0.326810000	-0.657008000
6	3.919238000	-0.031839000	-1.162459000
6	4.463500000	-1.279776000	-0.861628000
6	3.755767000	-2.168393000	-0.052720000
1	2.254048000	1.306206000	-0.910888000
1	4.469084000	0.665566000	-1.796575000
1	5.440745000	-1.560268000	-1.257188000
1	1.972190000	-2.514827000	1.093092000
1	4.178174000	-3.145668000	0.186625000

E = -698.032659564 au

ZPVE = 0.271339 au

Triphenylplumbyl radical – B3LYP/def2-TZVP

82	0.000000000	0.000000000	0.839651000
6	0.000000000	2.121319000	-0.011964000
6	-1.837116000	-1.060660000	-0.011964000
6	1.837116000	-1.060660000	-0.011964000
6	2.693278000	-0.387553000	-0.883588000
6	3.808966000	-1.032736000	-1.412322000
6	2.124037000	-2.379652000	0.339906000
6	3.239471000	-3.024521000	-0.188265000
6	4.082954000	-2.350976000	-1.065140000
1	2.495664000	0.640207000	-1.165934000
1	4.952247000	-2.850855000	-1.474084000
1	4.463506000	-0.503409000	-2.094623000
1	1.480877000	-2.920347000	1.025464000
1	3.449753000	-4.051232000	0.086951000
6	-1.011008000	2.526224000	-0.883588000
6	0.998821000	3.029296000	0.339906000
6	0.999576000	4.317725000	-0.188265000
6	-0.005472000	4.711429000	-1.065140000
6	-1.010107000	3.815029000	-1.412322000
1	-1.795788000	4.117215000	-2.094623000
1	-1.802267000	1.841205000	-1.165934000
1	1.788657000	2.742651000	1.025464000
1	1.783593000	5.013190000	0.086951000
1	-0.007211000	5.714199000	-1.474084000
6	-3.122857000	-0.649644000	0.339906000
6	-1.682270000	-2.138670000	-0.883588000
6	-2.798859000	-2.782293000	-1.412322000
6	-4.239048000	-1.293204000	-0.188265000
6	-4.077481000	-2.360454000	-1.065140000
1	-4.945036000	-2.863344000	-1.474084000
1	-2.667718000	-3.613805000	-2.094623000
1	-3.269534000	0.177697000	1.025464000
1	-5.233346000	-0.961958000	0.086951000
1	-0.693397000	-2.481412000	-1.165934000

E = -888.070808515 au

ZPVE = 0.267611 au

Triphenylplumbyl radical – B3LYP-D3(BJ)/def2-TZVP

82	0.000000000	0.000000000	0.934665000
6	0.000000000	2.084272000	0.015963000
6	-1.805032000	-1.042136000	0.015963000
6	1.805032000	-1.042136000	0.015963000
6	2.632169000	-0.335041000	-0.855347000
6	3.707698000	-0.969667000	-1.470564000
6	2.076474000	-2.383589000	0.279690000
6	3.151500000	-3.017641000	-0.335882000
6	3.967938000	-2.310360000	-1.211320000
1	2.437937000	0.709003000	-1.070891000
1	4.805790000	-2.802442000	-1.688585000
1	4.341179000	-0.415640000	-2.152497000
1	1.449997000	-2.948526000	0.960459000
1	3.351400000	-4.062335000	-0.131237000
6	-1.025930000	2.447046000	-0.855347000
6	1.026012000	2.990074000	0.279690000
6	1.037603000	4.238100000	-0.335882000
6	0.016862000	4.591515000	-1.211320000
6	-1.014092000	3.695794000	-1.470564000
1	-1.810635000	3.967392000	-2.152497000
1	-1.832983000	1.756814000	-1.070891000
1	1.828500000	2.729997000	0.960459000
1	1.842386000	4.933565000	-0.131237000
1	0.024092000	5.563157000	-1.688585000
6	-3.102486000	-0.606485000	0.279690000
6	-1.606239000	-2.112005000	-0.855347000
6	-2.693605000	-2.726127000	-1.470564000
6	-4.189104000	-1.220459000	-0.335882000
6	-3.984800000	-2.281155000	-1.211320000
1	-4.829881000	-2.760715000	-1.688585000
1	-2.530544000	-3.551752000	-2.152497000
1	-3.278497000	0.218529000	0.960459000
1	-5.193785000	-0.871230000	-0.131237000
1	-0.604954000	-2.465817000	-1.070891000

E = -888.153011052 au

ZPVE = 0.268019 au

Triphenylplumbyl radical – M06-2X/cc-pVDZ/SDD

82	0.000000000	0.000000000	-0.853114000
6	0.000000000	2.086472000	-0.001758000
6	-1.806938000	-1.043236000	-0.001758000
6	1.806938000	-1.043236000	-0.001758000
6	1.060276000	2.512384000	0.808996000
6	-2.705927000	-0.337966000	0.808996000
6	-2.055112000	-2.396388000	-0.267120000
6	-3.167386000	-3.037085000	0.280911000
6	-4.051686000	-2.326893000	1.092030000
6	-3.820174000	-0.977417000	1.355385000
1	-1.375614000	-2.969644000	-0.904077000
1	-3.344726000	-4.093342000	0.071391000
1	-4.923265000	-2.825645000	1.518330000
1	-4.509051000	-0.418150000	1.990732000
1	-2.536048000	0.719231000	1.031424000
6	1.645650000	-2.174418000	0.808996000
6	3.102889000	-0.581585000	-0.267120000
6	4.213886000	-1.224494000	0.280911000
6	2.756556000	-2.819659000	1.355385000
6	4.040991000	-2.345416000	1.092030000
1	3.259594000	0.293506000	-0.904077000
1	4.908713000	-2.850850000	1.518330000
1	5.217301000	-0.849946000	0.071391000
1	0.645152000	-2.555898000	1.031424000
1	2.616654000	-3.695878000	1.990732000
6	-1.047776000	2.977973000	-0.267120000
6	-1.046500000	4.261579000	0.280911000
6	0.010694000	4.672309000	1.092030000
6	1.063619000	3.797077000	1.355385000
1	-1.883981000	2.676138000	-0.904077000
1	-1.872576000	4.943288000	0.071391000
1	0.014552000	5.676495000	1.518330000
1	1.890896000	1.836667000	1.031424000
1	1.892397000	4.114028000	1.990732000

E = -698.057069354 au

ZPVE = 0.270504 au

6.3. Me_3T-TMe_3 Structures

Hexamethylethane – B3LYP-D3(BJ)/def2-TZVP

6	0.000000000	0.000000000	-0.000562000
6	0.000000000	1.459958000	-0.000402000
6	-0.989426000	2.195290000	-0.684709000
6	-0.984797000	3.580942000	-0.687438000
6	-0.000110000	4.284800000	0.000412000
6	0.984652000	3.580629000	0.687853000
6	0.989357000	2.194985000	0.684325000
1	-1.753189000	1.664510000	-1.236865000
1	-1.750029000	4.116241000	-1.236062000
1	-0.000174000	5.367330000	0.000733000
6	1.264361000	-0.729979000	-0.000402000
6	2.395890000	-0.240777000	-0.684709000
6	1.406234000	-1.954301000	0.684325000
6	2.608589000	-2.643048000	0.687853000
6	3.593586000	-0.937611000	-0.687438000
6	3.710801000	-2.142305000	0.000412000
1	4.648331000	-2.683514000	0.000733000
1	2.689376000	-3.573223000	1.236815000
1	2.318102000	0.686051000	-1.236865000
1	4.439784000	-0.542551000	-1.236062000
1	0.564471000	-2.350203000	1.236259000
6	-1.264361000	-0.729979000	-0.000402000
6	-2.395591000	-0.240684000	0.684325000
6	-1.406464000	-1.954513000	-0.684709000
6	-2.608788000	-2.643331000	-0.687438000
6	-3.593242000	-0.937581000	0.687853000
6	-3.710691000	-2.142495000	0.000412000
1	-2.317571000	0.686255000	1.236259000
1	-4.439189000	-0.542456000	1.236815000
1	-0.564913000	-2.350561000	-1.236865000
1	-4.648157000	-2.683816000	0.000733000
1	-2.689755000	-3.573690000	-1.236062000
1	1.753100000	1.663948000	1.236259000
1	1.749814000	4.115679000	1.236815000

E = -315.864001629 au

ZPVE = 0.244153 au

Hexamethyldisilane – B3LYP-D3(BJ)/def2-TZVP

14	0.000000000	0.000000000	1.173630000
14	0.000000000	0.000000000	-1.173630000
6	-0.890015000	-1.534571000	1.818074000
6	-0.883970000	1.538061000	1.818074000
6	1.773985000	-0.003490000	1.818074000
6	-1.773985000	-0.003490000	-1.818074000
6	0.890015000	-1.534571000	-1.818074000
6	0.883970000	1.538061000	-1.818074000
1	-1.920513000	1.571235000	1.475310000
1	-0.390874000	2.450365000	1.475208000
1	-0.893861000	1.555350000	2.911088000
1	2.317515000	-0.886676000	1.475208000
1	1.793903000	-0.003568000	2.911088000
1	2.320986000	0.877595000	1.475310000
1	-1.926641000	-1.563689000	1.475208000
1	-0.900042000	-1.551781000	2.911088000
1	-0.400473000	-2.448830000	1.475310000
1	0.390874000	2.450365000	-1.475208000
1	0.893861000	1.555350000	-2.911088000
1	1.920513000	1.571235000	-1.475310000
1	-2.317515000	-0.886676000	-1.475208000
1	-1.793903000	-0.003568000	-2.911088000
1	-2.320986000	0.877595000	-1.475310000
1	0.400473000	-2.448830000	-1.475310000
1	1.926641000	-1.563689000	-1.475208000
1	0.900042000	-1.551781000	-2.911088000

E = -818.741045404 au

ZPVE = 0.221681 au

Hexamethyldigermane – B3LYP-D3(BJ)/def2-TZVP

32	0.000000000	0.000000000	1.221497000
32	0.000000000	0.000000000	-1.221497000
6	-1.852259000	0.013698000	1.913959000
6	0.937992000	1.597254000	1.913959000
6	0.914267000	-1.610952000	1.913959000
6	-0.937992000	1.597254000	-1.913959000
6	-0.914267000	-1.610952000	-1.913959000
6	1.852259000	0.013698000	-1.913959000
1	0.449900000	2.508922000	1.568164000
1	1.974168000	1.610626000	1.574752000
1	0.931403000	1.594135000	3.005110000
1	0.407759000	-2.514992000	1.574752000
1	0.914860000	-1.603686000	3.005110000
1	1.947840000	-1.644085000	1.568164000
1	-2.381927000	0.904366000	1.574752000
1	-1.846263000	0.009551000	3.005110000
1	-2.397740000	-0.864836000	1.568164000
1	2.381927000	0.904366000	-1.574752000
1	1.846263000	0.009551000	-3.005110000
1	2.397740000	-0.864836000	-1.568164000
1	-1.974168000	1.610626000	-1.574752000
1	-0.931403000	1.594135000	-3.005110000
1	-0.449900000	2.508922000	-1.568164000
1	-1.947840000	-1.644085000	-1.568164000
1	-0.407759000	-2.514992000	-1.574752000
1	-0.914860000	-1.603686000	-3.005110000

E = -4393.77818740 au

ZPVE = 0.219193 au

Hexamethyldistannane – B3LYP-D3(BJ)/def2-TZVP

50	0.000000000	0.000000000	1.399180000
50	0.000000000	0.000000000	-1.399180000
6	-2.022282000	-0.076384000	2.191907000
6	0.944991000	1.789540000	2.191907000
6	1.077291000	-1.713156000	2.191907000
6	-0.944991000	1.789540000	-2.191907000
6	-1.077291000	-1.713156000	-2.191907000
6	2.022282000	-0.076384000	-2.191907000
1	0.413308000	2.678051000	1.853193000
1	1.980077000	1.849210000	1.857362000
1	0.926801000	1.760411000	3.281540000
1	0.611424000	-2.639402000	1.857362000
1	1.061160000	-1.682839000	3.281540000
1	2.112606000	-1.696961000	1.853193000
1	-2.591501000	0.790192000	1.857362000
1	-1.987961000	-0.077572000	3.281540000
1	-2.525914000	-0.981090000	1.853193000
1	2.591501000	0.790192000	-1.857362000
1	1.987961000	-0.077572000	-3.281540000
1	2.525914000	-0.981090000	-1.853193000
1	-1.980077000	1.849210000	-1.857362000
1	-0.926801000	1.760411000	-3.281540000

1	-0.413308000	2.678051000	-1.853193000
1	-2.112606000	-1.696961000	-1.853193000
1	-0.611424000	-2.639402000	-1.857362000
1	-1.061160000	-1.682839000	-3.281540000

E = -668.394324469 au

ZPVE = 0.215161 au

ZPVE = 0.231945 au

Hexamethyldiplumbane – B3LYP-D3(BJ)/def2-TZVP

82	0.000000000	0.000000000	1.456553000
82	0.000000000	0.000000000	-1.456553000
6	-2.086255000	-0.253322000	2.304436000
6	0.823744000	1.933410000	2.304436000
6	1.262510000	-1.680089000	2.304436000
6	-0.823744000	1.933410000	-2.304436000
6	-1.262510000	-1.680089000	-2.304436000
6	2.086255000	-0.253322000	-2.304436000
1	0.224183000	2.766404000	1.943964000
1	1.856567000	2.053067000	1.984566000
1	0.774334000	1.881683000	3.390778000
1	0.849724000	-2.634368000	1.984566000
1	1.242418000	-1.611434000	3.390778000
1	2.283685000	-1.577351000	1.943964000
1	-2.706292000	0.581301000	1.984566000
1	-2.016752000	-0.270248000	3.390778000
1	-2.507868000	-1.189054000	1.943964000
1	2.706292000	0.581301000	-1.984566000
1	2.016752000	-0.270248000	-3.390778000
1	2.507868000	-1.189054000	-1.943964000
1	-1.856567000	2.053067000	-1.984566000
1	-0.774334000	1.881683000	-3.390778000
1	-0.224183000	2.766404000	-1.943964000
1	-2.283685000	-1.577351000	-1.943964000
1	-0.849724000	-2.634368000	-1.984566000
1	-1.242418000	-1.611434000	-3.390778000

E = -625.452932580 au

ZPVE = 0.213134 au

Trimethyl(trimethylmethyl)silane – B3LYP-D3(BJ)/def2-TZVP

14	0.000000000	0.000000000	0.807138000
6	0.000000000	0.000000000	-1.102835000
6	0.000000000	-1.766792000	1.454350000
6	-1.530087000	0.883396000	1.454350000
6	1.530087000	0.883396000	1.454350000
6	-1.257199000	-0.709999000	-1.627214000
6	1.243477000	-0.733767000	-1.627214000
6	0.013722000	1.443766000	-1.627214000
1	-2.448809000	0.391838000	1.128155000
1	-1.569106000	1.922032000	1.119889000
1	-1.530773000	0.889753000	2.547031000
1	2.449082000	0.397870000	1.119889000
1	1.535935000	0.880812000	2.547031000
1	1.563746000	1.924812000	1.128155000
1	-0.879975000	-2.319902000	1.119889000
1	-0.005162000	-1.770564000	2.547031000
1	0.885063000	-2.316650000	1.128155000
1	-0.862882000	2.004910000	-1.296794000
1	0.012045000	1.450094000	-2.722658000
1	0.902500000	1.986924000	-1.299411000
1	-1.304862000	-1.749733000	-1.296794000
1	-1.261841000	-0.714615000	-2.722658000
1	-2.171977000	-0.211875000	-1.299411000
1	1.269477000	-1.775050000	-1.299411000
1	2.167744000	-0.255177000	-1.296794000
1	1.249795000	-0.735478000	-2.722658000

E = -567.309579734 au

6.4. Me_3T Radical Structures

(Trimethyl)methyl radical – B3LYP-D3(BJ)/def2-TZVP

6	0.000000000	0.000000000	0.156917000
6	0.000000000	1.479731000	-0.015233000
1	0.000000000	1.765985000	-1.080924000
1	-0.886118000	1.939466000	0.429244000
1	0.886118000	1.939466000	0.429244000
6	-1.281485000	-0.739866000	-0.015233000
1	-2.122686000	-0.202332000	0.429244000
1	-1.529388000	-0.882993000	-1.080924000
1	-1.236568000	-1.737134000	0.429244000
6	1.281485000	-0.739866000	-0.015233000
1	1.529388000	-0.882993000	-1.080924000
1	2.122686000	-0.202332000	0.429244000
1	1.236568000	-1.737134000	0.429244000

E = -157.874676692 au

ZPVE = 0.116131 au

(Trimethyl)silyl radical – B3LYP-D3(BJ)/def2-TZVP

14	0.000000000	0.000000000	0.430573000
6	0.000000000	1.784970000	-0.178663000
1	0.000000000	1.810441000	-1.274157000
1	-0.883010000	2.324349000	0.168398000
1	0.883010000	2.324349000	0.168398000
6	-1.545829000	-0.892485000	-0.178663000
1	-2.454451000	-0.397466000	0.168398000
1	-1.567888000	-0.905220000	-1.274157000
1	-1.571441000	-1.926884000	0.168398000
6	1.545829000	-0.892485000	-0.178663000
1	1.567888000	-0.905220000	-1.274157000
1	2.454451000	-0.397466000	0.168398000
1	1.571441000	-1.926884000	0.168398000

E = -409.308746662 au

ZPVE = 0.109213 au

(Trimethyl)germanyl radical – B3LYP-D3(BJ)/def2-TZVP

32	0.000000000	0.000000000	0.331336000
6	0.000000000	1.864912000	-0.346738000
1	0.000000000	1.846407000	-1.440087000
1	-0.886161000	2.399846000	-0.006871000
1	0.886161000	2.399846000	-0.006871000
6	-1.615061000	-0.932456000	-0.346738000
1	-2.521409000	-0.432485000	-0.006871000
1	-1.599035000	-0.923203000	-1.440087000
1	-1.635247000	-1.967362000	-0.006871000
6	1.615061000	-0.932456000	-0.346738000
1	1.599035000	-0.923203000	-1.440087000
1	2.521409000	-0.432485000	-0.006871000
1	1.635247000	-1.967362000	-0.006871000

E = -2196.83237463 au

ZPVE = 0.108086 au

(Trimethyl)stannyl radical – B3LYP-D3(BJ)/def2-TZVP

50	0.000000000	0.000000000	0.302882000
6	0.000000000	2.032317000	-0.511722000
1	0.000000000	1.959389000	-1.601219000
1	-0.888355000	2.572097000	-0.188245000
1	0.888355000	2.572097000	-0.188245000
6	-1.760038000	-1.016159000	-0.511722000
1	-2.671679000	-0.516711000	-0.188245000
1	-1.696880000	-0.979694000	-1.601219000
1	-1.783324000	-2.055386000	-0.188245000
6	1.760038000	-1.016159000	-0.511722000
1	1.696880000	-0.979694000	-1.601219000
1	2.671679000	-0.516711000	-0.188245000
1	1.783324000	-2.055386000	-0.188245000

E = -334.150198256 au

ZPVE = 0.106171 au

(Trimethyl)plumbyl radical – B3LYP-D3(BJ)/def2-TZVP

82	0.000000000	0.000000000	0.236086000
6	0.000000000	2.115271000	-0.667455000
1	0.000000000	1.985148000	-1.750016000
1	-0.893961000	2.645835000	-0.349142000
1	0.893961000	2.645835000	-0.349142000
6	-1.831878000	-1.057635000	-0.667455000
1	-2.738341000	-0.548725000	-0.349142000
1	-1.719189000	-0.992574000	-1.750016000
1	-1.844380000	-2.097110000	-0.349142000
6	1.831878000	-1.057635000	-0.667455000
1	1.719189000	-0.992574000	-1.750016000
1	2.738341000	-0.548725000	-0.349142000
1	1.844380000	-2.097110000	-0.349142000

E = -312.689321550 au

ZPVE = 0.104819 au

6.5. H_3T-TH_3 Structures

Ethane – B3LYP-D3(BJ)/def2-TZVP

6	-0.763102000	0.000000000	0.000000000
6	0.763102000	0.000000000	0.000000000
1	-1.160140000	1.001833000	-0.175684000
1	-1.160147000	-0.348768000	0.955453000
1	-1.160146000	-0.653062000	-0.779769000
1	1.160146000	0.653062000	0.779769000
1	1.160147000	0.348768000	-0.955453000
1	1.160140000	-1.001833000	0.175684000

E = -79.8694016893 au

ZPVE = 0.074443 au

Disilane – B3LYP-D3(BJ)/def2-TZVP

14	-1.172176000	-0.000001000	0.000001000
14	1.172176000	0.000001000	-0.000001000
1	-1.686684000	-1.302743000	0.487971000
1	-1.686703000	0.228773000	-1.372185000
1	-1.686694000	1.073969000	0.884215000
1	1.686696000	-1.073970000	-0.884212000
1	1.686703000	-0.228768000	1.372186000
1	1.686683000	1.302742000	-0.487975000

E = -582.651889852 au

ZPVE = 0.048925 au

Digermane – B3LYP-D3(BJ)/def2-TZVP

32	-1.217803000	0.000001000	0.000000000
32	1.217803000	-0.000001000	0.000000000
1	-1.754444000	1.358542000	-0.484790000
1	-1.754454000	-0.259434000	1.418920000
1	-1.754440000	-1.099124000	-0.934131000
1	1.754439000	1.099127000	0.934127000
1	1.754455000	0.259432000	-1.418920000
1	1.754445000	-1.358540000	0.484795000

E = -4157.71471215 au

ZPVE = 0.045797 au

Distannane – B3LYP-D3(BJ)/def2-TZVP

50	-1.393637000	-0.000001000	0.000000000
50	1.393637000	0.000001000	0.000000000
1	-2.000865000	1.276021000	-0.967936000
1	-2.000956000	0.200235000	1.588987000
1	-2.000837000	-1.476265000	-0.621120000
1	2.000830000	1.476267000	0.621121000
1	2.000957000	-0.200230000	-1.588987000
1	2.000870000	-1.276019000	0.967936000

E = -432.340974181 au

ZPVE = 0.039640 au

Diplumbane – B3LYP-D3(BJ)/def2-TZVP

82	-1.445442000	0.000000000	0.000000000
82	1.445442000	0.000000000	0.000000000
1	-2.075805000	-1.619927000	0.329503000
1	-2.075806000	0.524604000	-1.567638000
1	-2.075827000	1.095317000	1.238131000
1	2.075808000	-1.095278000	-1.238178000
1	2.075853000	-0.524642000	1.567607000
1	2.075805000	1.619937000	-0.329458000

E = -389.407464425 au

ZPVE = 0.036680 au

6.6. H_3T Radical Structures

Methyl radical – B3LYP-D3(BJ)/def2-TZVP

6	0.000000000	0.000000000	-0.000002000
1	0.539837000	-0.934179000	0.000003000
1	0.539105000	0.934602000	0.000003000
1	-1.078942000	-0.000423000	0.000003000

E = -39.8601344750 au

ZPVE = 0.029723 au

Silyl radical – B3LYP-D3(BJ)/def2-TZVP

14	0.000000000	-0.000001000	0.080286000
1	1.210576000	-0.725459000	-0.374667000
1	-1.233559000	-0.685649000	-0.374667000
1	0.022984000	1.411119000	-0.374664000

E = -291.265076033 au

ZPVE = 0.021195 au

Germanyl radical – B3LYP-D3(BJ)/def2-TZVP

32	0.000003000	0.000000000	0.041298000
1	-0.783815000	-1.238893000	-0.440510000
1	-0.681097000	1.298205000	-0.440506000
1	1.464827000	-0.059297000	-0.440536000

E = -2078.80091451 au

ZPVE = 0.019864 au

Stannyl radical – B3LYP-D3(BJ)/def2-TZVP

50	0.000000000	0.000001000	0.033074000
1	1.403229000	0.812840000	-0.551249000
1	-1.405594000	0.808742000	-0.551249000
1	0.002366000	-1.621647000	-0.551225000

E = -216.122990590 au

ZPVE = 0.017319 au

Plumbyl radical – B3LYP-D3(BJ)/def2-TZVP

82	0.000000000	0.000000000	0.022310000
1	1.456951000	-0.838500000	-0.609806000
1	-1.454641000	-0.842501000	-0.609806000
1	-0.002310000	1.680991000	-0.609810000

E = -194.664552504 au

ZPVE = 0.015745 au

7. Additional Literature

[1] M. J. Frisch, G. W. Trucks, H. B. Schlegel, G. E. Scuseria, M. A. Robb, J. R. Cheeseman, G. Scalmani, V. Barone, G. A. Petersson, H. Nakatsuji, X. Li, M. Caricato, A. V. Marenich, J. Bloino, B. G. Janesko, R. Gomperts, B. Mennucci, H. P. Hratchian, J. V. Ortiz, A. F. Izmaylov, J. L. Sonnenberg, Williams, F. Ding, F. Lipparini, F. Egidi, J. Goings, B. Peng, A. Petrone, T. Henderson, D. Ranasinghe, V. G. Zakrzewski, J. Gao, N. Rega, G. Zheng, W. Liang, M. Hada, M. Ehara, K. Toyota, R. Fukuda, J. Hasegawa, M. Ishida, T. Nakajima, Y. Honda, O. Kitao, H. Nakai, T. Vreven, K. Throssell, J. A. Montgomery Jr., J. E. Peralta, F. Ogliaro, M. J. Bearpark, J. J. Heyd, E. N. Brothers, K. N. Kudin, V. N. Staroverov, T. A. Keith, R. Kobayashi, J. Normand, K. Raghavachari, A. P. Rendell, J. C. Burant, S. S. Iyengar, J. Tomasi, M. Cossi, J. M. Millam, M. Klene, C. Adamo, R. Cammi, J. W. Ochterski, R. L. Martin, K. Morokuma, O. Farkas, J. B. Foresman, D. J. Fox, Wallingford, CT, **2016**.

[2] a) C. Lee, W. Yang, R. Parr, *Phys. Rev. B* **1988**, *37*, 785-789; b) A. D. Becke, *J. Chem. Phys* **1993**, *98*, 5648-5652.

[3] a) Y. Zhao, D. G. Truhlar, *Acc. Chem. Res.* **2008**, *41*, 157-167; b) Y. Zhao, D. G. Truhlar, *Theor. Chem. Acc.* **2008**, *120*, 215-241.

[4] a) S. Grimme, J. Antony, S. Ehrlich, H. Krieg, *J. Chem. Phys.* **2010**, *132*, 154104; b) S. Grimme, *WIREs Comput. Mol. Sci.* **2011**, *1*, 211-228.

[5] A. Schäfer, C. Huber, R. Ahlrichs, *J. Chem. Phys.* **1994**, *100*, 5829-5835.

[6] T. H. Dunning, *J. Chem. Phys.* **1989**, *90*, 1007-1023.

[7] a) E. R. Johnson, S. Keinan, P. Mori-Sánchez, J. Contreras-García, A. J. Cohen, W. Yang, *J. Am. Chem. Soc.* **2010**, *132*, 6498-6506; b) J. Contreras-García, E. R. Johnson, S. Keinan, R. Chaudret, J.-P. Piquemal, D. N. Beratan, W. Yang, *J. Chem. Theory Comput.* **2011**, *7*, 625-632.

[8] W. Humphrey, A. Dalke, K. Schulten, *J. Mol. Graph. Model.* **1996**, *14*, 33-38.

[9] a) W. B. Schneider, G. Bistoni, M. Sparta, M. Saitow, C. Riplinger, A. A. Auer, F. Neese, *J. Chem. Theory Comput.* **2016**, *12*, 4778-4792; b) A. Altun, F. Neese, G. Bistoni, *J. Chem. Theory Comput.* **2018**, *15*, 215-228; c) A. Altun, F. Neese, G. Bistoni, *Beilstein J. Org. Chem.* **2018**, *14*, 919-929.

[10] a) F. Neese, *WIREs Comput. Mol. Sci.* **2012**, *2*, 73-78; b) F. Neese, *WIREs Comput. Mol. Sci.* **2018**, *8*, e1327.

[11] a) S. E. Wheeler, K. N. Houk, P. v. R. Schleyer, W. D. Allen, *J. Am. Chem. Soc.* **2009**, *131*, 2547-2560; b) J. P. Wagner, P. R. Schreiner, *J. Chem. Theory Comput.* **2016**, *12*, 231-237.

[12] a) E. G. Hohenstein, C. D. Sherrill, *J. Chem. Phys.* **2010**, *133*, 014101; b) E. G. Hohenstein, R. M. Parrish, C. D. Sherrill, J. M. Turney, H. F. Schaefer, *J. Chem. Phys.* **2011**, *135*, 174107.

[13] a) J. M. Turney, A. C. Simmonett, R. M. Parrish, E. G. Hohenstein, F. A. Evangelista, J. T. Fermann, B. J. Mintz, L. A. Burns, J. J. Wilke, M. L. Abrams, N. J. Russ, M. L. Leininger, C. L. Janssen, E. T. Seidl, W. D. Allen, H. F. Schaefer, R. A. King, E. F. Valeev, C. D. Sherrill, T. D. Crawford, *WIREs Comput. Mol. Sci.* **2012**, *2*, 556-565; b) R. M. Parrish, L. A. Burns, D. G. A. Smith, A. C. Simmonett, A. E. DePrince, E. G. Hohenstein, U. Bozkaya, A. Y. Sokolov, R. Di Remigio, R. M. Richard, J. F. Gonthier, A. M. James, H. R. McAlexander, A. Kumar, M. Saitow, X. Wang, B. P. Pritchard, P. Verma, H. F. Schaefer, K. Patkowski, R. A. King, E. F. Valeev, F. A. Evangelista, J. M. Turney, T. D. Crawford, C. D. Sherrill, *J. Chem. Theory Comput.* **2017**, *13*, 3185-3197.

[14] T. M. Parker, L. A. Burns, R. M. Parrish, A. G. Ryno, C. D. Sherrill, *J. Chem. Phys.* **2014**, *140*, 094106.

[15] *Handbook of Chemistry and Physics*, 97th ed. (Ed.: W. M. Haynes), CRC Press., Cleveland, Ohio, **2016-2017**, p. 84.
[16] Y.-R. Luo, *Comprehensive handbook of chemical bond energies*, CRC press, **2007**.
[17] I. M. T. Davidson, A. V. Howard, *J. Chem. Soc., Farad. Trans. 1, Phys. Chem. Cond. Phases* **1975**, *71*, 69-77.
[18] T. Bernert, B. Winkler, Y. Krysiak, L. Fink, M. Berger, E. Alig, L. Bayarjargal, V. Milman, L. Ehm, P. W. Stephens, N. Auner, H.-W. Lerner, *Cryst. Growth Des.* **2014**, *14*, 2937-2944.
[19] M. Dräger, L. Ross, *Z. Anorg. Allg. Chem.* **1980**, *460*, 207-216.
[20] H. Preut, H. J. Haupt, F. Huber, *Z. Anorg. Allg. Chem.* **1973**, *396*, 81-89.
[21] a) H. Preut, H. Haupt, F. Huber, *Z. Anorg. Allg. Chem.* **1972**, *388*, 165-168; b) H. Preut, F. Huber, *Z. Anorg. Allg. Chem.* **1976**, *419*, 92-96.
[22] S. J. Blanksby, G. B. Ellison, *Acc. Chem. Res.* **2003**, *36*, 255-263.
[23] H. U. Buschhaus, W. P. Neumann, *Angew. Chem. Int. Ed.* **1978**, *17*, 59-59.
